# Ligand design by targeting a binding site water†

**DOI:** 10.1039/d0sc04938g

**Published:** 2020-11-19

**Authors:** Pierre Matricon, R. Rama Suresh, Zhan-Guo Gao, Nicolas Panel, Kenneth A. Jacobson, Jens Carlsson

**Affiliations:** Department of Cell and Molecular Biology, Science for Life Laboratory, Uppsala University SE-75124 Uppsala Sweden jens.carlsson@icm.uu.se; Molecular Recognition Section, Laboratory of Bioorganic Chemistry, National Institute of Diabetes and Digestive and Kidney Diseases, National Institutes of Health Bethesda Maryland 20892 USA

## Abstract

Solvent reorganization is a major driving force of protein–ligand association, but the contribution of binding site waters to ligand affinity is poorly understood. We investigated how altered interactions with a water network can influence ligand binding to a receptor. A series of ligands of the A_2A_ adenosine receptor, which either interacted with or displaced an ordered binding site water, were studied experimentally and by molecular dynamics simulations. An analog of the endogenous ligand that was unable to hydrogen bond to the ordered water lost affinity and this activity cliff was captured by molecular dynamics simulations. Two compounds designed to displace the ordered water from the binding site were then synthesized and evaluated experimentally, leading to the discovery of an A_2A_ agonist with nanomolar activity. Calculation of the thermodynamic profiles resulting from introducing substituents that interacted with or displaced the ordered water showed that the gain of binding affinity was enthalpy driven. Detailed analysis of the energetics and binding site hydration networks revealed that the enthalpy change was governed by contributions that are commonly neglected in structure-based drug optimization. In particular, simulations suggested that displacement of water from a binding site to the bulk solvent can lead to large energy contributions. Our findings provide insights into the molecular driving forces of protein–ligand binding and strategies for rational drug design.

## Abstract 2

Solvent reorganization is a major driving force of protein–ligand association, but the contribution of binding site waters to ligand affinity is poorly understood.

## Graphical abstract

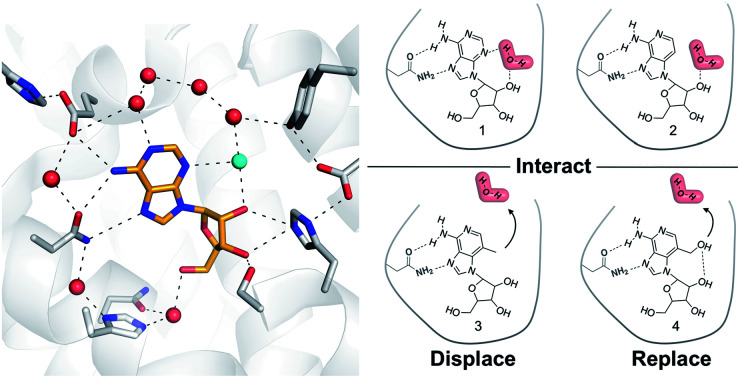

## Introduction

Understanding molecular recognition is one of the major goals of chemistry and biology. The determination of high-resolution structures of protein–ligand complexes gave birth to the idea of rationally designing drugs.^1^ Although a large number of theoretical approaches, ranging from empirical scoring functions^2^ to rigorous simulation methods,^3^ has been developed, none of these can consistently make accurate predictions of ligand affinities.^4,5^ The strength of a complex can be quantified based on the free energy of binding, which is determined by changes in enthalpy and entropy. The key to further improving computational models is to understand the link between molecular interactions and the thermodynamics of ligand binding by combining experimental and theoretical approaches. However, as the different energetic contributions to binding free energies are difficult to quantify, interpretation of structure–activity relationships can be a major challenge.^6,7^

Ligand binding to proteins is strongly affected by the aqueous environment. In fact, water displacement from the protein surface has been suggested to be a major driving force of ligand binding.^8^ In addition, ordered waters that bridge protein–ligand interactions are frequently observed in crystal structures and can contribute to ligand affinity.^9,10^ The possibility to exploit binding site waters in ligand optimization would be a valuable tool for rational drug design,^11^ but the consequences of perturbing hydration networks are poorly understood. Two main strategies are currently used to target water molecules in structure-based drug design. The first is to modify the chemical structure of a ligand to form hydrogen bonds with ordered waters in the protein–ligand interface. Consideration of such waters has been shown to improve the performance of scoring functions that predict ligand affinity.^12^ The second alternative is to displace water molecules by introducing a substituent that extends into a hydrated subpocket, which can yield large improvements of affinity.^13^ The appropriate substituent depends on the shape and polarity of the site, and the governing principle is that favorable protein–ligand interactions together with displacement of the water can lead to an energy gain. Molecular dynamics (MD) simulation and molecular docking studies support the importance of considering water displacement,^14–17^ but this energy term is difficult to predict and often neglected in practice. Intriguingly, water displacement can either be enthalpy or entropy driven, but the molecular details governing contributions to the free energy have only rarely been characterized in detail.^18–20^

**Fig. 1:**
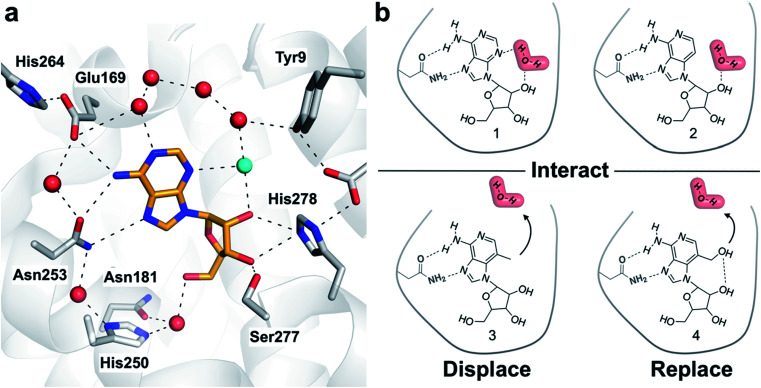
(a) Crystal structure of the A_2A_AR in complex with adenosine (PDB code: 2YDO).^22^ The receptor is depicted using white cartoons. Heavy atoms of adenosine and side chains that form key polar interactions are shown as sticks and hydrogen bonds are shown as black dashed lines. Crystal waters are represented as red spheres except the ordered water displaced by the designed ligands, which is colored in cyan. (b) Adenosine (1), 3-deazaadenosine (2), and two compounds (3 and 4) designed to probe how interactions with the binding site water network influence ligand binding.

Crystal structures of G protein-coupled receptors (GPCRs) have suggested that waters play important roles in ligand recognition in this family of important drug targets.^21^ A human A_2A_ adenosine receptor (A_2A_AR) structure demonstrated that the endogenous agonist (adenosine, 1) is stabilized by several waters (Fig. 1a).^22^ Structures of complexes with adenosine derivatives exemplify how ligand affinity can be improved by displacing ordered waters. One such water was found to form a hydrogen bond with the ribose 5′-hydroxyl group of adenosine. Early medicinal chemistry efforts discovered that potent agonists could be obtained by modifying the 5′-position of adenosine.^23^ A_2A_AR crystal structures determined 20 years later revealed that the 5′-*N*-ethyl-carboxamido substituent occupies the position of this water in the complex with adenosine and replaces its hydrogen bonds.^22^ We focused on a second ordered water in the A_2A_AR binding site that has not previously been targeted in agonist optimization. To assess the influence of modifying ligands to perturb hydration networks, compounds were designed to either interact with, displace, or replace the ordered water (Fig. 1b). MD simulations combined with free energy calculations were used to predict changes in affinity and characterize the thermodynamic consequences of altering interactions with binding site waters. Computational predictions were then evaluated experimentally for synthesized compounds. The results demonstrate how the combination of rigorous binding free energy calculations and analysis of hydration networks can be used to identify the driving forces of complex formation.

## Experimental section

### Molecular modelling

MD/FEP calculations were performed using the adenosine-bound A_2A_AR crystal structure (PDB code: 2YDO).^22^ The MD simulations were carried out with the program Q^24^ using the OPLS all atom force field^25^ in combination with implemented versions of the Berger lipids^26^ and the TIP3P water model.^27^ The OPLSAA force field combined with 1.14*CM1A-LBCC partial charges were used to parameterize compounds 1-4.^28^ The MD simulations of A_2A_AR–ligand complexes and ligands in aqueous solution were performed under spherical boundary conditions with a sphere radius of either 21 or 25 Å. Relative binding free energies were calculated based on the alchemical transformation of one ligand into another using a series of 84 intermediate states. Free energies were calculated using the Zwanzig equation and a thermodynamic cycle, as described previously.^29^ Hydration site analysis was carried out using GROMACS^30^ and SSTMap.^31^ Clustering in SSTMap was used to identify hydration sites, followed by calculation of enthalpies and entropies using the approach by Young *et al.*^8,16,31^ Molecular docking calculations were performed using AutoDock Vina^32^ and GLIDE.^33^ Detailed descriptions of the computational methods are available in the ESI.†

### Chemistry and biological assays

Compounds 3 and 4 were synthesized according to Schemes S1 and S2.† Radioligand binding assays were performed as previously described^34^ using membrane preparations from HEK293 cells stably expressing the human A_1_AR, A_2A_AR, A_2B_AR or A_3_AR. The agonist radioligands [^3^H](*R*)-*N*6-(phenylisopropyl)adenosine (R-PIA, 1 nM), [^3^H]2-[*p*-(2-carboxyethyl)phenylethylamino]-5′-*N*-ethylcarboxamidoadenosine (CGS21680, 8 nM), [^3^H]5′-*N*-ethylcarboxamidoadenosine (NECA, 25 nM), and [^125^I]*N*^6^-(4-amino-3-iodobenzyl)adenosine-5′-*N*-methyluronamide (I-AB-MECA, 0.2 nM) were used for the A_1_AR, A_2A_AR, A_2B_AR, and A_3_AR, respectively. Functional assays were carried out in HEK293 cells stably expressing a single hAR subtype. For determination of cAMP production, an ALPHAScreen cAMP kit (PerkinElmer) was used according to manufacturer's instructions. Detailed descriptions of the synthesis (including analytical data) and assays are available in the ESI.†

## Results

### Ligand design by targeting a binding site water

**Fig. 2:**
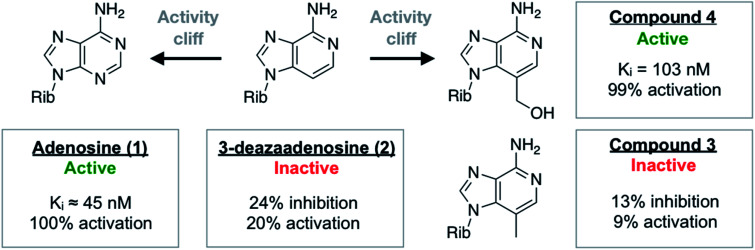
Summary of experimental data for adenosine analogs. For compounds 3 and 4, *K*_i_ values or percentage of inhibition at 10 μM was determined in a radioligand binding assay. All data are expressed as means resulting from three independent experiments. A_2A_AR activation (% activation) was determined in a functional assay measuring A_2A_AR-mediated stimulation of cAMP production at 10 μM. The experimental data are also summarized in Table S1.†

One of the most ordered waters in the crystal structure of the A_2A_AR–adenosine complex is trapped in a cavity where it forms hydrogen bonds to the adenine N3 atom, the ribose 2′-hydroxyl group, and a second crystal water (Fig. 1a). Three adenosine derivatives were designed to probe how perturbation of the binding site hydration network influenced ligand binding (Fig. 1b). The starting point of the study was the observation that 3-deazaadenosine (2), which lacks the N3 nitrogen that forms a hydrogen bond to the ordered water, was a weak ligand (Fig. 2 and Table S1†).^35^ In early work on adenosine receptors, this result was interpreted as if N3 was essential for activity and should be left unaltered.^36^ This could explain why there was not a single active adenosine derivative substituted with a carbon in this position among the >13 000 analogs tested at ARs in the ChEMBL bioactivity database.^37^ Moreover, we were intrigued by the fact that modification of a single heavy atom, which did not form any direct hydrogen bonds to the receptor, resulted in such a large loss of activity. The unexplored potential to target the ordered water led us to design two additional adenosine derivatives. The 3-methyl substituent of compound 3 was predicted to displace the water from the binding site whereas compound 4 was designed to displace the water and, in addition, replace its hydrogen bonds using a hydroxyl group (Fig. 1b).

### MD simulations predict large differences in ligand activity

The binding free energies (ΔΔ*G*_bind_) of adenosine, 3, and 4 relative to 3-deazaadenosine were predicted from MD simulations combined with rigorous free energy methods. MD simulations were performed in a spherical system centered on the binding site with explicit representation of protein, solvent, membrane, and ligand. The three compounds were alchemically transformed to 3-deazaadenosine in the receptor and in aqueous solution, and the free energy perturbation (FEP) technique was used to estimate the change in free energy with the program Q.^24^ Three sets of independent MD simulations were carried out for each transformation, resulting in a total of ∼300 ns per compound pair. A first set of simulations were performed for adenosine and 3-deazaadenosine to benchmark if the simulation protocol could capture the experimentally observed activity cliff. Transformation of the C3 position to a nitrogen resulted in a relative binding free energy of −6.7 kcal mol^−1^. This result was consistent with the large affinity reduction observed experimentally for 3-deazaadenosine. The precise value of the magnitude of the experimental difference is uncertain because 3-deazaadenosine has very low affinity (39 μM) and it is difficult to measure the affinity of adenosine in standard binding assays because adenosine deaminase is added to degrade endogenous adenosine. An approximate *K*_i_ value of adenosine obtained from assays carried out without addition of adenosine deaminase is 45 nM,^22^ which would correspond to a free energy difference of −4.0 kcal mol^−1^. The activities of compounds 3 and 4 were then predicted using the same MD simulation protocol. Introduction of the 3-methyl and 3-hydroxymethyl groups on the 3-deazaadenosine scaffold to displace the ordered water resulted in ΔΔ*G*_FEP_ values of −2.2 and −5.8 kcal mol^−1^, respectively. These results indicated that compound 4 would be an agonist with similar activity as adenosine whereas 3 would be a considerably weaker ligand.

### Synthesis of adenosine derivatives

Several routes were explored to prepare the target nucleosides 3 and 4. By the first route, the protected nucleobase 3,3,4,4-tetramethyl-1-(7-methyl-1*H*-imidazo[4,5-*c*]pyridin-4-yl)pyrrolidine-2,5-dione 7 was prepared from 7-methyl-1*H*-imidazo[4,5-*c*]pyridin-4-amine 5 according to a literature procedure (Scheme S1†).^38^ The protected sugar moiety 1-*O*-acetyl-2,3,5-tri-*O*-benzoyl-d-ribofuranose 8 was coupled with 7 using standard Vorbrüggen reaction conditions. Thus, compound 7 was presilylated with *N*,*O*-bis(trimethylsilyl)acetamide (BSA) in acetonitrile and treated with 8 in the presence of TMSOTf to give 9 with complete regioselectivity. It is noteworthy that the tetramethylsuccinoyl (M_4_S) protecting group shields the *N*^3^ and *N*^5^ nucleophilic centers of 7 (corresponding to adenosine *N*^7^ and *N*^1^, respectively) and can lead to only *N*^1^-glycosylation of purines (adenosine *N*^9^), as observed by others.^38^ Compound 9 was then converted to target compound 3 by the debenzoylation with saturated NH_3_ in MeOH at 80 °C in a sealed tube. The reaction time for the complete deprotection was 40 h. The benzoyl groups were removed during 20 h reflux, but the M_4_S group remained as indicated on TLC (higher polarity than 9) and by mass spectrometry, in contrast to faster deprotection with similar conditions as reported by Arico *et al.*^38^ Therefore, the reaction was continued for an additional 20 h at 80 °C to afford the complete deprotection to 3. Compound 3 was also obtained using an alternative route (Scheme S2†). The reaction of 9 with *N*-bromosuccinimide in the presence of a catalytic amount of benzoyl peroxide led to the corresponding mono-brominated derivative (10), which was treated with sodium acetate (NaOAc) in DMF to give the *O*-acetyl derivative 11 in good yield (81%). This acetate derivative 11 was refluxed with saturated NH_3_ in MeOH for 36 h in a sealed tube to obtain target compound 4. More detailed synthetic procedures are described in the ESI.†

### Biological activity of adenosine derivatives

The activities of adenosine and 3-deazaadenosine at the A_2A_AR were available from previous work.^22,35^ For compounds 3 and 4, both binding and functional assays at the A_2A_AR were carried out (Fig. 2). Experiments were also performed for the A_1_, A_2B_, and A_3_ subtypes (Fig. S1 and Table S1†). Whereas compound 3 did not show significant displacement of radioligand at 10 μM, compound 4 was a high affinity A_2A_AR ligand with a *K*_i_ value of 103 nM. Similarly, measurement of G_s_-mediated cAMP production in response to the compounds showed that compound 4 was a full agonist (EC_50_ = 732 nM) of this receptor whereas compound 3 was inactive. In agreement with the MD/FEP calculations, compound 4 was hence a potent agonist with similar affinity as adenosine. Compound 3 was also correctly predicted to be less active than adenosine. The difference in activity between 3 and 3-deazaadenosine (2) could not be determined precisely due to their low affinity. Further experiments demonstrated that compound 4 also activated the A_1_ and A_3_ subtypes, but not the A_2B_AR (Table S1†). The resistance of compound 4 to degradation by adenosine deaminase was also concluded qualitatively by comparing its measured A_2A_AR activity in the absence and presence of adenosine deaminase, which makes this compound an interesting scaffold for development of AR agonists.

### Activity cliffs originate from changes in binding enthalpy

**Fig. 3:**
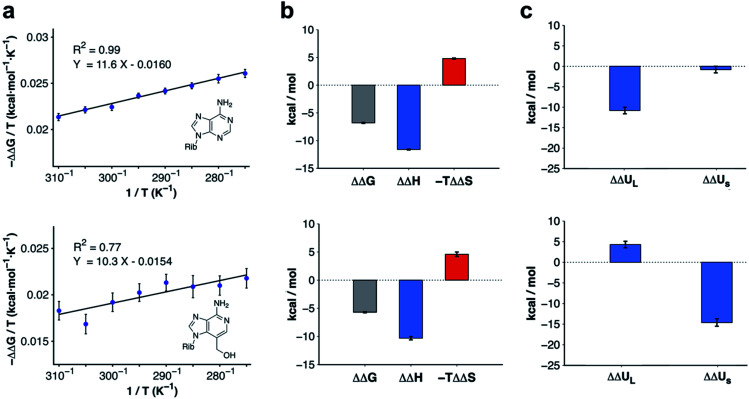
Thermodynamic profiles for adenosine and compound 4. (a) Computational van't Hoff plots based on binding free energies relative to 3-deazaadenosine calculated at different temperatures for adenosine and compound 4. (b) Thermodynamic profiles show that the relative free energy change at 300 K for adenosine and compound 4 is driven by enthalpy. (c) Decomposition of the relative binding enthalpy for adenosine and compound 4 into contributions from the ligand and from within the surroundings. The ligand contribution is the driving force for adenosine whereas changes from within the surroundings dominate for compound 4. The calculated energies and uncertainties in (b) and (c) are also shown in Table S2.†

Modifications to 3-deazaadenosine by either introducing an N3 or 3-hydroxymethyl group led to large improvements of ligand affinity. As these activity cliffs were captured by the MD/FEP calculations, we performed additional simulations to elucidate the thermodynamic and molecular basis of these differences. First, changes in enthalpy (ΔΔ*H*) and entropy (ΔΔ*S*) compared to 3-deazaadenosine were estimated from a van't Hoff analysis.^39^ Based on relative binding free energies calculated by MD/FEP at eight temperatures, ΔΔ*H* and ΔΔ*S* could be determined from the slope and intercept of a linear regression of Δ*G*/*T versus* 1/*T* (Fig. 3a and Table S3†). The total MD simulation time to generate one van't Hoff plot was ∼2–4 μs, which yielded converged ΔΔ*H* and ΔΔ*S* values with uncertainties <0.5 kcal mol^−1^ (Table S2†). In both cases, a large enthalpy gain was responsible for the improvement of activity, which was counteracted by an entropy loss (Fig. 3b and Table S2†).

The calculations for adenosine resulted in ΔΔ*H* and −*T*ΔΔ*S* values equal to −11.6 ± 0.1 and 4.8 ± 0.1 kcal mol^−1^ at 300 K, respectively. For compound 4, the corresponding enthalpy and entropy contributions were −10.3 ± 0.3 and 4.6 ± 0.4 kcal mol^−1^, respectively. Control calculations using a larger simulation sphere radius of 25 Å demonstrated that the thermodynamic profiles were independent of system size (Table S3,† ΔΔ*H* and −*T*ΔΔ*S* equal to −11.0 ± 0.1 and 4.5 ± 0.1 kcal mol^−1^ for adenosine, and −12.7 ± 0.1 and 8.0 ± 0.2 kcal mol^−1^ for compound 4). Based on these results, the activity cliffs arising from introducing a hydrogen bond to a water with adenosine or displacing it from the binding site with compound 4 had similar thermodynamic origins.

### Molecular basis of enthalpy driven binding

To identify the structural basis of the large enthalpy gains for adenosine and compound 4 relative to 3-deazaadenosine, this term was decomposed into contributions from the ligand (ΔΔ*U*_L_, representing ligand–receptor, ligand–water, and ligand strain energies) and from within the surroundings (ΔΔ*U*_S_, representing energy from interactions between receptor, membrane, and water atoms). As the pressure–volume contribution to the enthalpy is negligible, the enthalpy can be approximated as the sum of these two energies (ΔΔ*H* ≈ ΔΔ*U*_L_ + ΔΔ*U*_S_). Extended MD simulations of the ligands in water and bound to the receptor allowed us to calculate ΔΔ*U*_L_ (Table S4†).^40^ In contrast, ΔΔ*U*_S_ would be too difficult to converge from brute-force simulations due to the large number of interactions contributing to this term. However, precise values of ΔΔ*U*_S_ could be estimated indirectly by subtracting the difference in ligand energy from the ΔΔ*H* extracted from van't Hoff analysis (ΔΔ*U*_S_ ≈ ΔΔ*H* − ΔΔ*U*_L_). The improvement of binding enthalpy for adenosine (relative to 3-deazaadenosine) was due to changes in ligand energy (ΔΔ*U*_L_ = −10.8 ± 0.8 kcal mol^−1^) and the contribution from within the surroundings was small (ΔΔ*U*_S_ = −0.8 ± 0.8 kcal mol^−1^). In contrast, the balance between ΔΔ*U*_L_ and ΔΔ*U*_S_ was inverted for compound 4. The interaction energy from within the surroundings (ΔΔ*U*_S_ = −14.6 ± 0.9 kcal mol^−1^) dominated and the change in ligand enthalpy was unfavorable (ΔΔ*U*_L_ = 4.3 ± 0.8 kcal mol^−1^). The decomposition of enthalpy contributions hence demonstrated that the similar gains of affinity for adenosine and compound 4 had different molecular origins (Fig. 3c).

### Ligand enthalpy explains activity cliff for adenosine

Ligand contributions to the relative binding enthalpy (ΔΔ*U*_L_) were identified based on the MD simulation trajectories. Analysis of the energetics revealed that the difference in enthalpy between adenosine and 3-deazaadenosine was not primarily driven by hydrogen bonding to the binding site water network (Table S4†). Approximately half of the ΔΔ*U*_L_ term was due to more favorable receptor–ligand complementarity (−3.6 ± 0.6 kcal mol^−1^) and ligand internal energy (−1.7 ± 0.3 kcal mol^−1^). There was also a lower desolvation term for adenosine (−5.5 ± 0.8 kcal mol^−1^), but this contribution was dominated by differences in ligand–water interaction energy for the unbound state rather than from the complex. The difference in ligand–water interaction energy for the unbound state was 6.5 ± 0.2 kcal mol^−1^ whereas the corresponding value for the complex was only 1.0 ± 0.8 kcal mol^−1^. The enthalpy difference for compound 4 relative to 3-deazaadenosine was also favoured by receptor–ligand interaction energies (−8.4 ± 0.6 kcal mol^−1^) and the ligand internal energy (−4.7 ± 0.5 kcal mol^−1^). However, these terms could not compensate for the large desolvation penalty (17.5 ± 0.8 kcal mol^−1^). Notably, the potent compound 4 would be predicted to be inactive based solely on the relative ligand energy of 4.3 ± 0.8 kcal mol^−1^. Instead, changes from within the surroundings were the driving force of the improved binding in that case.

### Water–water enthalpy explains activity cliff for compound 4

The molecular basis of the enthalpy changes from within the surroundings could not be quantified without further approximations due to the large fluctuations in this energy. Considering the close similarity of the ligands and the small structural differences between the receptor structures for the different complexes in MD simulations, we hypothesized that contributions from the ligands in aqueous environment would rather be small and that instead hydration network reorganization in the binding site would dominate ΔΔ*U*_S_. This idea was reinforced by the enthalpic term estimated from van't Hoff analysis being maintained even if the receptor was restrained to the crystal coordinates in the MD simulations (Table S3,† ΔΔ*H* equal to −12.7 ± 0.1 and −13.5 ± 0.4 for adenosine and compound 4, respectively).

**Fig. 4:**
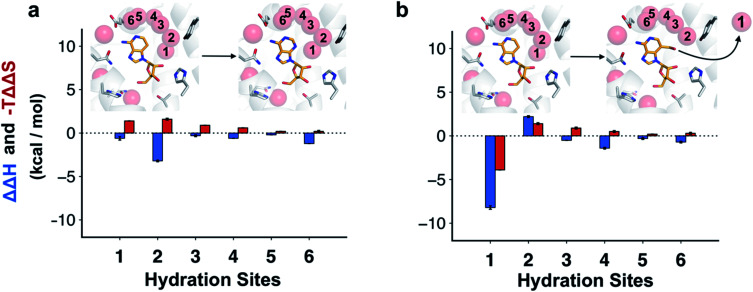
Changes in structure and energy of the hydration network. Hydration sites identified by MD simulations of the A_2A_AR bound to (a) adenosine and (b) compound 4. The receptor is depicted using white cartoons. Each hydration site is shown as a transparent red sphere. The heavy atoms of the compounds are shown in sticks with orange carbons. Key binding site residues are shown with white carbons. Each hydration site is labelled with a number and the corresponding difference in interaction energy of the surrounding and entropy relative to 3-deazaadenosine were calculated using inhomogeneous solvation theory (blue and red bars, respectively).

To assess how different ligands perturbed the water network, MD simulations in which the protein and ligand heavy atoms were restrained to the crystal coordinates were performed. Snapshots of the water network were then clustered to identify highly occupied hydration sites.^31^ In the case of adenosine, the hydration site interacting with the N3 atom was observed in 100% of the MD snapshots, confirming that the water was highly ordered (Fig. 4a). An additional five waters with high occupancies were identified in the vicinity of the adenine moiety and these were supported by the electron density of the crystal structure.^22^ For 3-deazaadenosine, the ordered water interacting with the N3 of adenosine remained in the same site in 78% of the MD snapshots despite that it lost one hydrogen bond to the ligand. Furthermore, the hydration network structure was otherwise identical to that obtained with adenosine. The ordered water was completely displaced from the binding site in simulations of compound 4, but the remaining water network was again very similar to that obtained with adenosine (Fig. 4b).

Water interaction energies and approximate entropies for hydration sites were analyzed using inhomogeneous solvation theory.^31,41^ Enthalpy and entropy changes from within the surroundings (*i.e.* water–water and water–protein interactions) were estimated for each of the six hydration sites interacting with the adenine moiety (Fig. 4). The hydration sites for compound 4 and 3-deazaadenosine were then compared. The two hydration sites that were closest to the 3-hydroxymethyl and C3 substituents had the largest changes in entropy and enthalpy (Fig. 4b), but the energies of the entire network were influenced. In particular, displacement of the ordered water led to a large decrease of the enthalpy of the surroundings (−8.2 kcal mol^−1^). Of this term, −12.7 kcal mol^−1^ originated from water–water interactions, which was counteracted by +4.5 kcal mol^−1^ from protein–water interactions. These results agreed qualitatively with the MD/FEP calculations, which predicted an enthalpic contribution of −14.6 kcal mol^−1^ from within the surroundings. Interestingly, water displacement also led to a large entropy contribution of −3.9 kcal mol^−1^ relative to 3-deazaadenosine, but as the remaining waters became more ordered, the resulting total contribution to the free energy change was small. Whereas adenosine binding was primarily driven by changes in ligand energy, the enthalpy from the water network reorganization was crucial to capture the activity cliff observed experimentally for compound 4. In the case of adenosine, there was a smaller decrease of the enthalpy of the surroundings and this term was largely counteracted by a large unfavorable entropy term of similar magnitude (Fig. 4a).

### Molecular docking scoring functions fail to predict activity cliffs

To assess the added value of performing MD simulations compared to less computationally demanding methods, compound affinities were predicted with the molecular docking programs GLIDE^33^ and AutoDock Vina^32^ (Table S5†). The receptor binding site was prepared using default protocols and the sampling settings were optimized to reproduce the binding mode of the adenosine scaffold. The GLIDE-SP scoring function yielded overall similar scores for all compounds and predicted that 3-deazaadenosine was more potent than adenosine. AutoDock Vina also predicted that adenosine had a lower affinity than 3-deazaadenosine and that the weak compound 3 was the most potent ligand. Hence, none of these docking programs were able to predict the activity cliffs observed experimentally for adenosine and compound 4.

## Discussion

Water plays important roles in molecular recognition, but the energetic consequences of interaction and displacement of binding site water molecules are poorly understood. In this work, we probed how ordered waters could be targeted in ligand optimization. MD simulations combined with compound synthesis and biological assays led to three main findings. First, large changes in activity due to small chemical modifications of ligand structure were captured by MD/FEP calculations, which led to the discovery of an unanticipated, potent A_2A_AR agonist. Second, we demonstrated that the molecular basis of such activity cliffs can be deduced by partitioning the calculated free energy into enthalpic and entropic components. Third, the simulations showed that binding affinity changes were the result of a complex interplay between energy terms that are commonly neglected in rational drug design. Whereas traditional structure-based approaches focus on protein–ligand interactions, MD simulations revealed that large increases of activity can be driven by changes in ligand strain, ligand desolvation, water–water interactions.

One of the fundamental assumptions of ligand-based drug design is that similar molecules will have similar biological activity. Such methods will fail to recognize when a small change to a ligand structure leads to a large change in affinity.^42^ Physics-based models have the potential to predict receptor–ligand binding affinities accurately and are gaining interest as a tool for drug design.^3^ Computational identification of activity cliffs could reduce the resource demand associated with hit optimization. In the case of the A_2A_AR, MD/FEP calculations captured the loss of binding due to modification of a single heavy atom in adenosine to create 3-deazaadenosine. With such compounds, MD/FEP is perfectly suited to probe different substituents. Encouraged by the computational predictions, we pursued multi-step synthesis of compound 4, which was verified to be a full agonist equipotent to adenosine.

From a more fundamental viewpoint, we were interested in understanding the driving forces behind the activity cliffs. As the free energy reflects enthalpic and entropic changes, we reasoned that insights could be made based on these components. Such thermodynamic profiling has also been proposed as a tool to guide drug optimization efforts.^6,43^ To obtain thermodynamic signatures, we used computational van't Hoff analysis, which has previously been applied to understand host–guest systems,^44^ ion hydration,^39^ and temperature adaptation of enzymes.^45^ The use of classical MD simulations allowed us to disentangle enthalpic contributions from different molecular interactions using force field energies, with initially surprising results. Despite adenosine and compound 4 being closely related analogs, the observed activity cliffs had different molecular origins. For adenosine, we anticipated the affinity change to be driven by hydrogen bonding to the ordered water. Although the interaction with the ordered water may be important for the absolute binding affinity of adenosine, interactions with water in the complex could not explain the change in affinity compared to 3-deazaadenosine. Instead, contributions from induced fit, ligand strain, and hydration of the unbound ligand played more prominent roles. These three terms are often neglected by empirical scoring functions^2^ and, indeed, two widely-used docking programs failed to predict the activity cliffs. Similar observations were made by Leung *et al.* in a study of why addition of methyl substituents can lead to activity cliffs. Whereas affinity gains of methyl substituents are commonly attributed to the hydrophobic effect, a reduced ligand conformational energy penalty was also found to make important contributions to binding.^46^ These results emphasize the importance of considering both the bound and unbound states of a compound in the lead optimization process, which generally is focused mainly on receptor–ligand interactions. In contrast to adenosine, an enthalpy gain originating from water displacement was shown to be essential for predicting the observed activity cliff for compound 4. The contribution from ligand interactions, which is the only term considered by many scoring functions, was even unfavorable. This result again highlighted the complexity of predicting drug binding using simplified models. As rational drug design is heavily biased toward ligand interactions, indirect optimization routes by optimizing water–water or protein–water interaction networks are left unexplored.

Water reorganization is one of the principal driving forces of ligand binding, but is still very challenging to predict. In the classical view based on the hydrophobic effect, water displacement from a binding site leads to a gain of entropy due to the release of constrained waters into the bulk solvent. However, changes in binding free energy due to capture or release of waters can also be completely enthalpy driven. These scenarios and the tremendous influence of hydration networks on ligand binding have been demonstrated in a number of experimental studies.^6,47^ The dynamical nature of interactions with water molecules in the solute–solvent interface complicates analysis of hydration networks based on MD simulations. Although hydration site analysis based on a rigid receptor structure involves several approximations,^31^ this approach can provide valuable insights into the structure and thermodynamics of water networks.^48–50^ In the case of adenosine, no water was displaced and the introduced ligand–water hydrogen bond was counterbalanced by an entropy loss due to rigidification of the solvent network. This exemplifies entropy–enthalpy compensation and suggests that it will be difficult to increase affinity by introducing hydrogen bonds to binding site waters. Moreover, the fact that perturbation of a single water influenced the energetics of the entire hydration network shows that it will be challenge to develop simplified descriptions of binding site hydration. Compound 4 illustrated that if ligand binding involves release or capture of water molecules, large gains of energy can be obtained. Neglecting this contribution to the free energy inventory will lead to large prediction errors.

## Conclusions

Prediction of protein–ligand binding affinities is the holy grail of computer-aided drug design. However, free energies by themselves, irrespective if they originate from experimental or computational sources, do not provide the molecular interpretation that may be necessary to identify candidates for the next round of drug optimization. The unique approach taken here makes it possible to link energetics to interactions, which has the potential to accelerate the discovery of therapeutic agents.

## Author contributions

P. M., K. A. J., and J. C. designed research; P. M. performed MD simulations, free energy calculations, and hydration site analysis; R. R. S. performed synthesis and characterization of compounds; Z.-G. G. performed biological assays; N. P. performed molecular docking calculations; P. M, R. R. S., Z.-G. G, and N. P. analyzed data; all authors contributed to the writing of the paper.

## Conflicts of interest

There are no conflicts to declare.

## Supplementary Material

SC-012-D0SC04938G-s001
