# Supplementary material for: Ligand design by targeting a binding site water
Source: Chem Sci. 2020 Nov 19;12(3):960–8. doi: 10.1039/d0sc04938g (PMC8179138; doi:10.1039/d0sc04938g)
Supplement: SC-012-D0SC04938G-s001 [file SC-012-D0SC04938G-s001.pdf]

Electronic Supplementary Material (ESI) for Chemical Science.
This journal is © The Royal Society of Chemistry 2020

Supplementary Information

Ligand design by targeting a binding site water

Pierre Matricon,[‡a] R. Rama Suresh,[‡b] Zhan-Guo Gao,[b] Nicolas Panel,[a] Kenneth A. Jacobson,[b] and Jens Carlsson[*a]

[a] Department of Cell and Molecular Biology, Science for Life Laboratory, Uppsala University, SE-75124 Uppsala, Sweden

[b] Molecular Recognition Section, Laboratory of Bioorganic Chemistry, National Institute of Diabetes and Digestive and Kidney Diseases, National Institutes of Health, Bethesda, Maryland 20892, USA

[‡] These authors contributed equally to this work.

[*]E-mail: jens.carlsson@icm.uu.se

Table of Contents

**Supplementary figures and tables**	
Figure S1	S3
Tables S1-S5	S4-8
**Supplementary methods**	S9-12
**Supplementary figures, results and methods for chemistry**	S13-27
**Analytical spectra and structural assignment of synthesized compounds**	S28-41
**Supplementary analytical figures**	S42-53
**Supplementary references**	S54

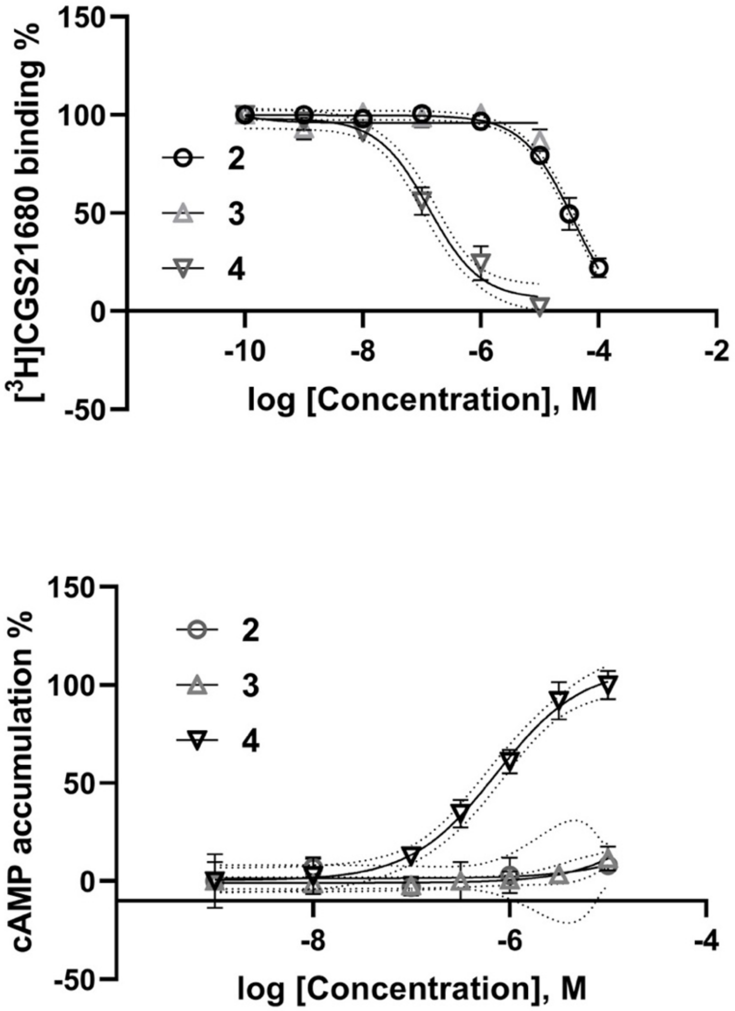

Figure S1. Inhibition of $A_{2A}AR$ radioligand binding (upper panel) and stimulation of $A_{2A}AR$ dependent cAMP accumulation (lower panel) for compounds **2**-**4**. Experimental procedures are described in the methods section.

Table S1. Pharmacological data (binding K_i and functional EC_{50} values) at human ARs for compounds **1-4** (n=3).

Cmpd	K_i (nM) or *% Inhibition at 10µM*[a]				EC_{50} (nM) and *Maximum efficacy*[a]			
	A_1	A_{2A}	A_{2B}	A_3	A_1	A_{2A}	A_{2B}	A_3
**1**	-	45[b]	-	-	310[c]	730<br>*100%*[c]	23,500[c]	290[c]
**2**	461 ± 19[d]	39,000<br>*(24 ± 8%)*[d]	-	*53 ± 11%*[d]	-	*20 ± 4%*	-	-
**3**	*48 ± 17%*	*13 ± 1%*	*1 ± 1%*	*50 ± 2%*	-	*9 ± 1%*	-	-
**4**	31 ± 4	103 ± 25	29 ± 7%	280 ± 27	180 ± 38<br>*101 ± 3%*	732 ± 95<br>*99 ± 3%*	*<15%*	318 ± 66<br>*97 ± 4%*

[a]All data are expressed as mean ± standard error resulting from three independent experiments. Experimental proceedures are described in the methods section. [b]Value from Lebon *et al.*[1] [c]Values from Fredholm *et al.*[2] [d]Values from Rodriguez *et al.*[3]

Table S2. Summary of thermodynamic profiles of adenosine and compound **4** calculated from MD simulations (kcal/mol).

	van't Hoff analysis[a]			Enthalpic decomposition[a]	
Cmpd	ΔΔG	ΔΔH	–TΔΔS	$\Delta\Delta U_L$[b]	$\Delta\Delta U_S$[c]
**Adenosine**	–6.8 ± 0.1	–11.6 ± 0.1	4.8 ± 0.1	–10.8 ± 0.8	–0.8 ± 0.8
**4**	–5.7 ± 0.1	–10.3 ± 0.3	4.6 ± 0.4	4.3 ± 0.8	–14.6 ± 0.9

[a]ΔΔH and ΔΔS values were determined using MD/FEP from the slope and intercept of a linear regression of ΔΔG/T versus 1/T (Fig. 3a). The ΔΔG values are averages calculated based on three independent sets of MD/FEP calculations at 300 K. Uncertainties for ΔΔH and –TΔΔS at 300 K were calculated as the SEM from eight values obtained from leave-one-out analysis of the van't Hoff plot. Potential energy values are averages ± SEM from 10 independent simulations (1 ns each). [b]Relative ligand potential energy representing ligand-receptor, ligand-water, and ligand-ligand interaction energies. [c]The potential energy from within the surroundings ($\Delta\Delta U_S$) was calculated from the difference between the total enthalpy (ΔΔH) and the ligand enthalpy ($\Delta\Delta U_L$).

Table S3. Data from binding free energy calculations using MD/FEP (kcal/mol). All energies were calculated relative to 3-deazaadenosine (**2**). Entropies and enthalpies were obtained using van’t Hoff analysis.

<table>
<tr><th>Cmpd</th><th>Simulation sphere radius (Å)</th><th>Restrained Protein</th><th>$\Delta\Delta G^{a}$</th><th>$\Delta\Delta H^{b}$</th><th>$-T\Delta\Delta S^{b}$</th></tr>
<tr><td rowspan="3">1</td><td rowspan="2">21</td><td>No</td><td>–6.8 ± 0.1</td><td>–11.6 ± 0.1</td><td>4.8 ± 0.1</td></tr>
<tr><td>Yes</td><td>–9.2 ± 0.1</td><td>–12.7 ± 0.1</td><td>3.5 ± 0.1</td></tr>
<tr><td>25[c]</td><td>No</td><td>–6.5 ± 0.0</td><td>–11.0 ± 0.1</td><td>4.5 ± 0.1</td></tr>
<tr><td rowspan="3">4</td><td rowspan="2">21</td><td>No</td><td>–5.7 ± 0.1</td><td>–10.3 ± 0.3</td><td>4.6 ± 0.4</td></tr>
<tr><td>Yes</td><td>–6.1 ± 0.1</td><td>–13.5 ± 0.4</td><td>7.4 ± 0.4</td></tr>
<tr><td>25[c]</td><td>No</td><td>–4.7 ± 0.1</td><td>–12.7 ± 0.1</td><td>8.0 ± 0.2</td></tr>
<tr><td rowspan="2">3</td><td>21</td><td>No</td><td>–2.0 ± 0.1</td><td>–10.3 ± 0.2</td><td>8.3 ± 0.2</td></tr>
<tr><td>25[c]</td><td>No</td><td>–1.9 ± 0.1</td><td>–8.4 ± 0.2</td><td>6.5 ± 0.2</td></tr>
</table>

[a]The $\Delta\Delta G$ values are averages calculated based on three independent sets of MD/FEP calculations at 300 K. [b]$\Delta\Delta H$ and $\Delta\Delta S$ values were determined using MD/FEP from the slope and intercept of a linear regression of $\Delta\Delta G/T$ versus $1/T$. Uncertainties for $\Delta\Delta H$ and $-T\Delta\Delta S$ at 300 K were calculated as the SEM from eight values obtained from leave-one-out analysis of the van’t Hoff plot. [c]MD simulations performed with doubled production time compared to the 21 Å system.

Table S4. Decomposition of $\Delta\Delta U_L$ for adenosine and compound **4** obtained from MD simulations.

Cmpd	Ligand energy terms (kcal/mol)[a]			Total
	ligand-water	protein-ligand	ligand internal	
**Adenosine**				
$U_{L,complex}$	–21.3 ± 0.4	–82.3 ± 0.1	–5.6 ± 0.1	
$U_{L,water}$	–92.8 ± 0.1	-	–6.4 ± 0.2	
**2**				
$U_{L,complex}$	–22.3 ± 0.7	–78.7 ± 0.6	18.1 ± 0.1	
$U_{L,water}$	–99.3 ± 0.1	-	15.6 ± 0.2	
**4**				
$U_{L,complex}$	–18.4 ± 0.2	–87.2 ± 0.2	22.9 ± 0.3	
$U_{L,water}$	–113.1 ± 0.3	-	25.1 ± 0.3	
**Adenosine**[b]				
$\Delta U_{L,complex}$	1.0 ± 0.8	–3.6 ± 0.6	–23.7 ± 0.2	
$\Delta U_{L,water}$	6.5 ± 0.2	-	–22.0 ± 0.3	
$\Delta\Delta U_L$	–5.5 ± 0.8	–3.6 ± 0.6	–1.7 ± 0.3	–10.8 ± 0.8
**4**[b]				
$\Delta U_{L,complex}$	3.8 ± 0.7	–8.4 ± 0.6	4.7 ± 0.3	
$\Delta U_{L,water}$	–13.7 ± 0.3	-	9.5 ± 0.4	
$\Delta\Delta U_L$	17.5 ± 0.8	–8.4 ± 0.6	–4.7 ± 0.5	4.3 ± 0.8

[a]Energy values are averages ± standard error of the mean from data obtained with 10 independent simulations (1 ns each) for adenosine and compound **4** and 20 simulations for compound **2**. [b]Values are relative to compound **2**.

Table S5. Relative binding energy scores for adenosine, compound **3**, and **4** using GLIDE-SP and AutoDock Vina. All energies are calculated relative to 3-deazaadenosine.

	Docking score (kcal/mol)	
Cmpd	GLIDE-SP	AutoDock Vina
**Adenosine**	0.4	0.2
**3**	–0.1	–0.6
**4**	–0.1	–0.2

Supplementary methods

MD simulations and free energy calculations. All MD simulations were performed based on the atomic coordinates of the adenosine-bound A_{2A}AR crystal structure (PDB code: 2YDO)[1]. Engineered mutations were reverted, and the pseudo-apo receptor was placed in a pre-equilibrated 1-palmitoyl-2-oleoyl phosphatidylcholine (POPC) lipid bilayer using the GPCR-ModSim protocol[4]. These calculations were performed under periodic boundary conditions in GROMACS[5]. The system was equilibrated for 20 ns at 310 K and atmospheric pressure using the OPLS 2005 all atom force field[6], Berger lipids[7], and the TIP3P water model[8]. The receptor structure was held rigid using tight positional restraints on protein heavy atoms whereas the membrane and water molecules were allowed to relax. Each receptor-ligand complex was then prepared based on a snapshot of the equilibrated system. The binding modes of compounds **2**-**4** were modelled based on the A_{2A}AR-adenosine complex and waters overlapping with the ligands were removed. The MD/FEP calculations were performed with Q[9] using the OPLS force field implemented for this program[10]. The simulations were performed under spherical boundary conditions with a sphere radius of 21 Å centered on the ligand (unless noted otherwise). The OPLSAA_2005 force field from the ffld_server program (Schrödinger, LLC, New York, NY, 2017) combined with 1.14*CM1A-LBCC partial charges were used to parameterize compounds **1**-**4**[11]. Atoms outside the sphere were excluded from non-bonded interactions. Ionizable residues close to the sphere edge were set to their neutral form and atoms within 3 Å of the sphere edge were restrained to their initial coordinates. Water molecules at the sphere border were subject to radial and polarization restraints according to the surface-constrained all atom solvent (SCAAS) model[12]. Solvent bonds and angles were constrained

using the SHAKE algorithm[13]. A cutoff of 10 Å was used for non-bonded interactions except for ligand atoms, for which no cutoff was applied. Long-range electrostatic interactions were treated using the local reaction field approximation[14]. Protonation states of ionizable residues Asp, Glu, Arg, and Lys in the binding site were set to the most probable in aqueous solution at pH 7. His75 and His155 were protonated at the δ position whereas His250 was protonated at the ε position. His264 and His278 were protonated at both the δ and ε positions. In all simulations, a time step of 1 fs was used and non-bonded pair lists were updated every 25 steps. Relative binding free energies were calculated based on the alchemical transformation of one ligand into another in a series of 84 intermediate states and three replicas per window. The transformations were divided into four major steps: (A) Transformation of partial charges, (B) introduction of soft-core potentials on atoms to be annihilated[15], (C) transformation of Lennard-Jones parameters for annihilated of atoms, (4) transformation of bonded terms and remaining Lennard-Jones parameters. The four steps were typically performed with 11, 11, 21 and 41 intermediate states, respectively. At each intermediate state, the receptor-ligand complexes were first equilibrated for 475 ps. During the equilibration, harmonic positional restraints on solute heavy atoms were gradually released and the system was heated to the target temperature. This was followed by a 250 ps production phase and energies were collected every 50 fs. Each ligand was also prepared for simulations in aqueous solution using a water droplet of the same size. In this case, the equilibration and production phase times were 350 ps and 100 ps, respectively. In these simulations, a harmonic restraint was applied to a central ligand atom to prevent it from approaching the sphere edge. Free energies were calculated using the Zwanzig equation and a thermodynamic cycle, as described previously[16].

Hydration site analysis were carried out using GROMACS [5] and SSTMap[17] using the same force field as for the MD/FEP calculations. Three replicas of each complex were simulated with solute heavy atoms tightly restrained to their initial coordinates. The systems were first heated to 300 K in the NVT ensemble, which was followed by a 1 ns equilibration step in the NPT ensemble. Subsequently, three independent NVT simulations of 10 ns of each complex were performed and 10,000 snapshots were collected. Clustering in SSTMap was used to identify ordered waters, followed by calculation of enthalpies and entropies for these hydration sites using the approach by Young *et al.*[17–19].

Molecular Docking. Docking calculations with AutoDock Vina were performed using default settings in a 15 Å box centered on the ligand[20]. The GLIDE docking calculations were carried out using the OPLS3e force field and GLIDE-SP scoring function[21]. In the GLIDE calculations, the ring puckering of adenosine was fixed in order to obtain the same binding mode as that observed the adenosine-bound $A_{2A}AR$ crystal structure[1].

Biological Assays. Radioligand binding assays were performed as previously described[22] using membrane preparations from HEK293 cells stably expressing the human A_1AR, $A_{2A}AR$, $A_{2B}AR$ or A_3AR. The agonist radioligands [^{3}H](*R*)-*N*^{6}-(phenylisopropyl)adenosine (R-PIA, 1 nM), [^{3}H]2-[*p*-(2-carboxyethyl)phenylethylamino]-5′-*N*-ethylcarboxamidoadenosine (CGS21680, 8 nM), [^{3}H]5′-N-ethylcarboxamidoadenosine (NECA, 25 nM) and [^{125}I]*N*^{6}-(4-amino-3-iodobenzyl)adenosine-5′-*N*-methyluronamide (I-AB-MECA, 0.2 nM) were used for the A_1AR, $A_{2A}AR$, $A_{2B}AR$ and A_3AR, respectively. Non-specific binding was determined

using 8-[4-[[[[(2-aminoethyl)amino]carbonyl]methyl]oxy]phenyl]-1,3-dipropylxanthine (XAC) at 10 μM for A_1 and A_{2A} and 100 μM for A_{2B} and A_3. Binding parameters were calculated using Prism 8 software (GraphPAD, San Diego, CA, USA). IC_{50} values obtained from competition curves were converted to K_i values using the Cheng–Prusoff equation[23]. Data are expressed as mean ± standard error.

Functional assays were carried out in HEK293 cells stably expressing a single hAR subtype. The cells were cultured in DMEM supplemented with 10% fetal bovine serum, 100 units/mL penicillin, 100 μg/mL streptomycin, and 2 μmol/mL glutamine. Cells were plated in 96-well plates in 100 μL of medium. After overnight, the medium was removed, and the cells were washed three times with 100 μL of DMEM containing 50 mM HEPES (pH 7.4). For A_{2A}AR agonist activity assessment, cells were treated for 20 min with agonists in the presence of rolipram (10 μM) and adenosine deaminase (3 units/mL). For other subtypes, cells were first incubated with agonists for 20 min, and forskolin (10 μM) was then added, after which the mixture was incubated for an additional 15 min. To test antagonist activity, compounds were added 20 min before the addition of agonists. The reactions were terminated by supernatant removal, and cells were lysed upon the addition of 100 μL of lysis buffer (0.3% Tween-20). For determination of cAMP production, an ALPHAScreen cAMP kit (PerkinElmer) was used according to manufacturer's instructions.

Supplementary Figures, Results and Methods for Chemistry

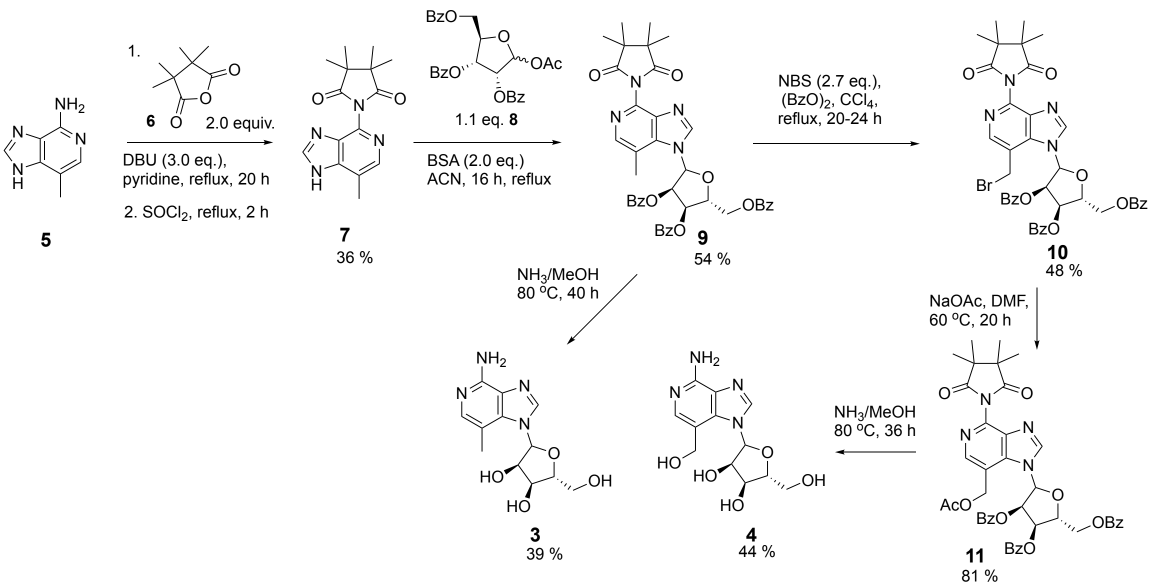

Scheme S1 Synthesis of compounds **3** and **4**.

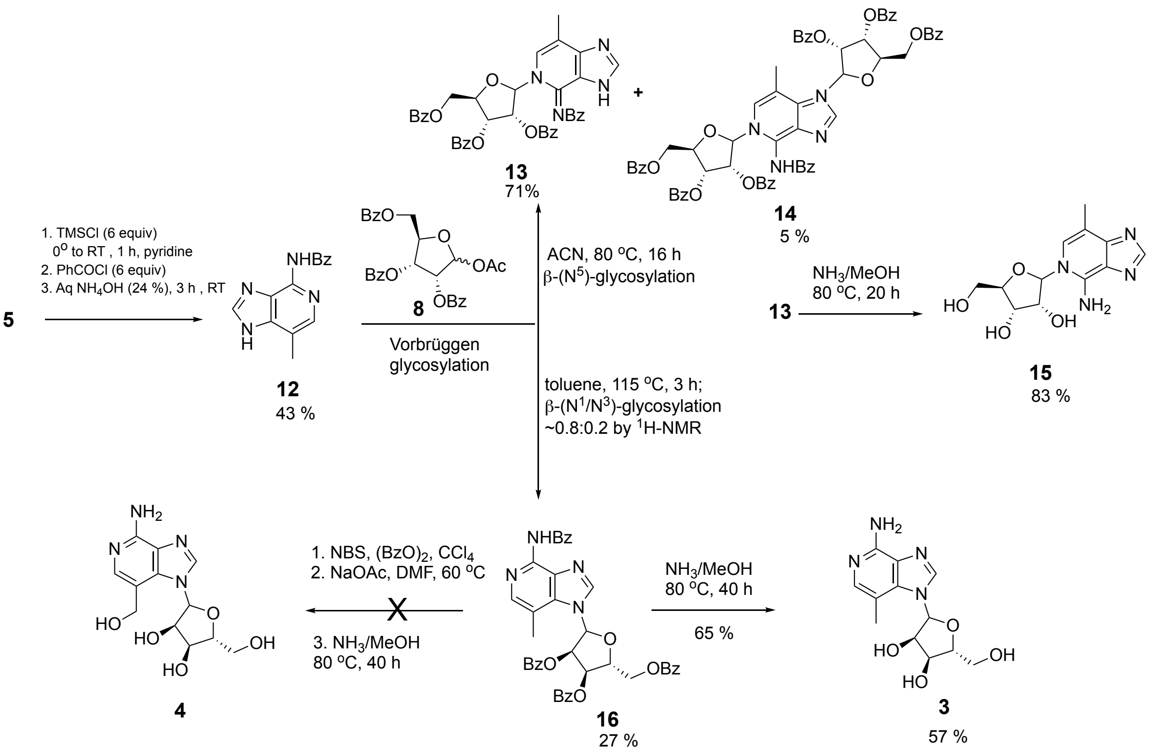

Scheme S2 Alternative synthesis of compound **3**.

Alternative synthesis route for compound 3. As described in Scheme S2, the amino group of 7-methyl-1H-imidazo[4,5-c]pyridin-4-amine **5** was protected with benzoyl chloride in the presence of TMS-Cl to afford the corresponding protected derivative **12** in 43% yield (slightly higher than with M4S). Compound **12** was subjected to a Vorbrüggen coupling with **8** using TMSOTf to give the corresponding undesired β-N^5-ribosylated derivative/riboside (**13**, kinetic product). Initially, we expected that the coupling product would be the more stable β-N^1-ribosylated derivative (**16**, thermodynamic product), which was difficult to differentiate from **13** by its mass and ^{1}H-NMR spectrum in chloroform-d. Consequently, the riboside derivative **13** was treated with sat. NH_3 in MeOH for 20 h, and the product **15** was isolated as a white solid. However, subsequent analysis revealed that the product was neither the N^1- nor the N^3- ribosylated derivative, but the β-N^5-ribosylated derivative **15**. A similar observation was reported for the ribosylation of adenine by

Framski *et al.*[24]. The assigned structure was confirmed by NMR. The absence of the N^1-addition product under these conditions was probably due to the shielding of the N^1-position by the pyridine 7-methyl group.

When the solvent was changed from acetonitrile to toluene, the more stable thermodynamic product, N^1-ribosylated derivative **16**, could be obtained at reflux temperature (115°C) predominantly along with N^3-isomer (N^1:N^3 ratio ~0.8:0.2, by ^{1}H-NMR). The NMR spectra are in good agreement with the assigned structure, as is evident using NOE, HMBC and ^{2}H-isotopic shift. The final assignment of the configuration of **16** was confirmed by the deprotection of benzoyl groups with sat. NH_3/MeOH at 80 °C for 40 h to give the desired riboside **3**, which was fully characterized by NMR (HSQC, HMBC, 1D-NOE).

Several attempts were made to brominate the methyl group in compound **16** using the conditions as used for compound **9**, but no desired mono-bromo compound could be isolated, although a product peak was observed by mass spectrometry. We did not optimize the reaction conditions for the bromination of **16**. Had we been able to isolate the desired bromo derivative, compound **4** could have been prepared by this route. Based on these results, it is emphasized that the Vorbrüggen reaction regioselectivity with protected forms of **5** depends upon the solvent, temperature and the protecting group/substituent on N^4-atom. The overall yield of compound **3** using *N*-benzoyl protection was 7.6%, and we feel that overall yield could be improved by optimizing reaction conditions. However, the more general synthetic approach proved to be using the M_4S protecting group.

General comments. 7-Methyl-1*H*-Imidazo[4,5-c]pyridin-4-amine (**5**) was purchased from AstaTech (Bristol, PA, USA). Other reagents and solvents were purchased from Millipore Sigma (St. Louis, MO, USA). The NMR spectra were recorded using a 400, 500 or 600 MHz spectrometer in $CDCl_3$ (7.26 ppm), CD_3OD (HOD = 4.87 ppm) or in $(CD_3)_2SO$ (^1H=2.50 ppm and ^{13}C=39.52 ppm). Confirmation of the product structures were obtained by mass spectrometry and standard 1D and 2D NMR methods including HSQC, and HMBC. ^{13}C-isotope shift experiments were acquired after adding 1 μL of D_2O to the sample tube. NOE contacts were established with a double pulsed field gradient spin-echo (dpfgse) pulse sequence using 30 ms Q3 Gaussian cascade selective refocusing pulses. HMBC experiments used the standard Bruker *hmbcetgpl2nd* sequence. TLC analysis was carried out on glass sheets precoated with silica gel F254 (0.2 mm) from Aldrich. The purity of final nucleoside derivatives (**3** and **4**) was checked using a Hewlett−Packard 1100 HPLC equipped with an Agilent Eclipse 5 μm XDB-C18 analytical column (50 mm × 4.6 mm; Agilent Technologies Inc., Palo Alto, CA). Mobile phase: linear gradient solvent system, 10 mM TEAA (triethyllammonium acetate) −CH_3CN from 95:5 to 0:100 in 20 min; the flow rate was 1.0 mL/min. Peaks were detected by UV absorption with a diode array detector at 230, 254, and 280 nm. All derivatives tested for biological activity showed >95% purity in the HPLC systems. Low-resolution mass spectrometry was performed with a JEOL SX102 spectrometer with 6 kV Xe atoms following desorption from a glycerol matrix or on an Agilent LC/MS 1100 MSD, with a Waters Atlantis C18 column (Milford, MA, USA). High resolution mass spectroscopic (HRMS) measurements were performed on a proteomics optimized Q-TOF-2 (Micromass-Waters) using external calibration with polyalanine, unless noted. Mass accuracies were observed.

The compounds 2,2,3,3-tetramethylsuccinic anhydride (**6**)[2], 3,3,4,4-tetramethyl-1-(7-methyl-1*H*-imidazo[4,5-c]pyridin-4-yl)pyrrolidine-2,5-dione (**7**), and *N*-(7-methyl-1*H*-imidazo[4,5-c]pyridin-4-yl)benzamide (**12**) were prepared according to literature procedures[25].

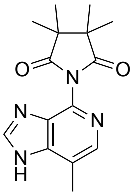

7

3,3,4,4-Tetramethyl-1-(7-methyl-1*H*-imidazo[4,5-c]pyridin-4-yl)pyrrolidine-2,5-dione (7):

Yield: 0.079 g (36%; brown solid; using 0.768 mmol of 7-methyl-1*H*-imidazo[4,5-*c*]pyridin-4-amine, **5**).

^{1}H NMR (400 MHz, chloroform-*d*) δ 8.04 (s, 1H), 7.41 (s, 1H), 2.28 (s, 3H), 1.39 (s, 12H).

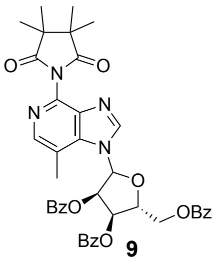

9

(2*R*,3*R*,4*R*)-2-((Benzoyloxy)methyl)-5-(7-methyl-4-(3,3,4,4-tetramethyl-2,5-dioxopyrrolidin-1-yl)-1*H*-imidazo[4,5-*c*]pyridin-1-yl)tetrahydrofuran-3,4-diyl dibenzoate (9):

Bis(trimethylsilyl)acetamide (BSA, 111 µl, 92 mg, 0.454 mmol, 2.0 equiv.) was added to a stirred solution of **7** (65 mg, 0.227mmol, 1.0 equiv.) and 1-*O*-acetyl-2,3,5-tri-*O*-benzoyl-D-ribofuranose (**8**) (126 mg, 0.250 mmol, 1.1 equiv.) in dried acetonitrile (4 mL), and stirring was continued under N_2 at 50 °C for 20 min. To this clear solution, TMSOTf (45 µL, 0.250 mmol, 1.1 equiv.) was added, and the reaction mixture stirred at gentle reflux, under N_2 for 16 h. The solvent was removed using a rotary evaporator to obtain the residue. The crude was extracted with ethyl acetate (20 mL) and washed with saturated $NaHCO_3$ (10 mL). The layers were separated, and the aqueous phase was washed with ethyl acetate (20 mL). The combined organic phase was washed with brine (20 mL), dried over anhydrous Na_2SO_4, filtered, and concentrated using a rotary evaporator. The crude product was purified by column chromatography (hexane/EtOAc; 1:1.5) to give the protected nucleoside **9** (90 mg, 54%) as a white solid. The eluant was 40-50 % EtOAc in hexane.

^{1}H NMR (400 MHz, chloroform-*d*) δ 8.25 (d, *J* = 17.5 Hz, 2H), 8.11 (d, *J* = 7.7 Hz, 2H), 7.96 (d, *J* = 7.8 Hz, 2H), 7.91 (d, *J* = 7.8 Hz, 2H), 7.58 (dq, *J* = 13.4, 7.1 Hz, 3H), 7.49 (s, 1H), 7.47 – 7.33 (m, 3H), 7.26 (s, 0H), 6.68 (d, *J* = 6.6 Hz, 1H), 6.13 (t, *J* = 6.2 Hz, 1H), 5.97 (t, *J* = 4.4 Hz, 1H), 4.86 – 4.75 (m, 2H), 4.69 (dd, *J* = 12.4, 3.5 Hz, 1H), 2.76 (s, 3H), 1.35 (s, 12H).

HRMS (ESI) m/z: $[M+H]^+$ calculated for $C_{41}H_{39}N_4O_9$: 731.2717; found 731.2705.

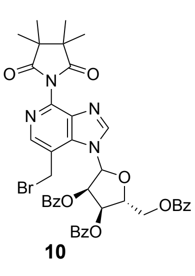

10

(2*R*,3*R*,4*R*)-2-((Benzoyloxy)methyl)-5-(7-(bromomethyl)-4-(3,3,4,4-tetramethyl-2,5-dioxopyrrolidin-1-yl)-1*H*-imidazo[4,5-*c*]pyridin-1-yl)tetrahydrofuran-3,4-diyldibenzoate (10): To a stirred solution of riboside (**9,** 80 mg, 0.109 mmol, 1.0 equiv.) in CCl_4 (5 mL), *N*-bromosuccinimide (31 mg, 0.1744 mmol, 1.60 equiv.) and benzoic anhydride ($(BzO)_2$, 1.32 mg, 0.05 equiv.) were added and the reaction mixture was stirred under N_2 at 80 °C for 16 h. A small aliquot was examined by mass spectrometry, showed some starting material (**9**). Additional 1.2 equiv. of NBS and 0.1 equiv. of $(BzO)_2$ were added and the reaction mixture was stirred for 6 h at 75 °C. The solvent was evaporated using a rotary evaporator. The residue was diluted with DCM (30 mL), washed with saturated $NaHCO_3$ (10 mL), brine (10 mL), dried over anhydrous Na_2SO_4, and filtered, and the solvent was removed using a rotary evaporator. The crude product was purified by column chromatography to give the bromo derivative **10** (42 mg, 48%) as a white solid. The eluant was 35-40 % EtOAc in hexane.

^{1}H NMR (400 MHz, chloroform-*d*) δ 8.41 (d, *J* = 1.6 Hz, 1H), 8.29 (d, *J* = 1.6 Hz, 1H), 8.12 (d, *J* = 7.6 Hz, 3H), 8.09 – 8.01 (m, 2H), 7.87 (d, *J* = 7.8 Hz, 2H), 7.61 (t, *J* = 7.5 Hz, 2H), 7.49 (ddt, *J* = 24.7, 15.0, 7.5 Hz, 2H), 7.34 (t, *J* = 7.7 Hz, 2H), 7.03 (dd, *J* = 7.4, 1.6 Hz, 1H), 6.32 (t, *J* = 6.4 Hz, 1H), 6.05 (dt, *J* = 4.9, 2.1 Hz, 1H), 5.07 (d, *J* = 11.1 Hz, 1H), 4.91 – 4.73 (m, 3H), 4.66 (dd, *J* = 12.9, 3.7 Hz, 1H), 1.35 (s, 12H).

HRMS (ESI) m/z: $[M+H]^+$ calculated for $C_{41}H_{38}N_4O_9{}^{79}Br$ 809.1822; found 809.1807.

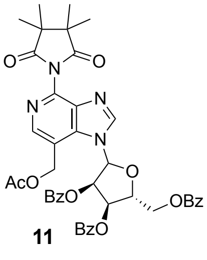

11

(3*R*,4*R*,5*R*)-2-(7-(Acetoxymethyl)-4-(3,3,4,4-tetramethyl-2,5-dioxopyrrolidin-1-yl)-1*H*-imidazo[4,5-*c*]pyridin-1-yl)-5-((benzoyloxy)methyl)tetrahydrofuran-3,4-diyl dibenzoate (11): To a stirred solution of riboside (**7,** 38 mg, 0.047 mmol, 1.0 equiv.) in DMF (5 mL), sodium acetate (30 mg, 0.376 mmol, 8.0 equiv.) was added and the reaction mixture was stirred under N_2 at 70 ºC for 20 h. The solvent was evaporated using a rotary evaporator. The residue was diluted with ethyl acetate (20 mL), washed with water (10 mL), brine (10 mL), dried over anhydrous Na_2SO_4, and filtered, and the solvent was removed using a rotary evaporator. The crude product was purified by column chromatography to give the acetate derivative **11** (30 mg, 81%) as a white solid. The eluant was 60 % EtOAc in hexane.

^{1}H NMR (400 MHz, chloroform-*d*) δ 8.48 (s, 1H), 8.32 (s, 1H), 8.09 (d, *J* = 7.8 Hz, 2H), 8.00 (d, *J* = 7.3 Hz, 3H), 7.89 (d, *J* = 7.8 Hz, 2H), 7.58 (dt, *J* = 21.4, 7.6 Hz, 3H), 7.46 (dt, *J* = 20.7, 7.6 Hz, 3H), 7.36 (t, *J* = 7.7 Hz, 2H), 6.65 (d, *J* = 6.8 Hz, 1H), 6.23 (t, *J* = 6.2 Hz, 1H), 5.99 (dd, *J* = 5.7, 3.2 Hz, 1H), 5.60 – 5.48 (m, 2H), 4.83 (t, *J* = 3.3 Hz, 1H), 4.76 (dd, *J* = 12.4, 2.9 Hz, 1H), 4.67 (dd, *J* = 12.4, 3.7 Hz, 1H), 2.03 (s, 3H), 1.35 (s, 12H).
HRMS (ESI) m/z: $[M+H]^+$ calculated for $C_{43}H_{41}N_4O_{11}$ 789.2772; found 789.2764.

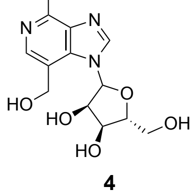

4

(3*R*,4*S*,5*R*)-2-(4-Amino-7-(hydroxymethyl)-1*H*-imidazo[4,5-*c*]pyridin-1-yl)-5-(hydroxymethyl)tetrahydrofuran-3,4-diol (4): In a 25 mL sealed tube were added

compound **11** (30 mg, 0.038 mmol) and freshly prepared saturated ammonia in MeOH (5.0 mL). The mixture was sealed and the reaction mixture stirred at 80 ^oC for 36 h. The solvent was removed and co-evaporated with ethanol. The crude product was purified by column chromatography to give the compound **4** (5.0 mg, 44%) as a white solid. The eluant was 10-20 % MeOH in dichloromethane (DCM) with 5% aq. NH_3 (23%).

^1H NMR (400 MHz, Methanol-d_4) δ 8.61 (t, J = 3.4 Hz, 1H), 7.66 (s, 1H), 6.44 (dt, J = 6.5, 3.3 Hz, 1H), 4.81 – 4.70 (m, 1H), 4.40 – 4.25 (m, 2H), 4.17 – 4.08 (m, 1H), 3.97 – 3.83 (m, 1H), 3.84 – 3.69 (m, 1H).

HRMS (ESI) m/z: $[M+H]^+$ calculated for $C_{12}H_{17}N_4O_5$ 297.1199; found 297.1201.

Purity by analytical HPLC: > 98%. Rt: 3.31 min.

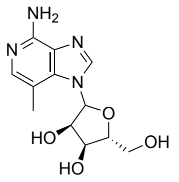

3

(3*R*,4*S*,5*R*)-2-(4-Amino-7-methyl-1*H*-imidazo[4,5-*c*]pyridin-1-yl)-5-(hydroxymethyl)tetrahydrofuran-3,4-diol (3) from compound 9: In a 25 mL sealed tube were added compound (**9**) (20 mg, 0.0273 mmol) and saturated ammonia in MeOH (5.0 mL). The mixture was sealed and the reaction mixture stirred at 80 ^oC for 36 h. The solvent was removed and co-evaporated with ethanol. The crude product was purified by column chromatography to give the compound **3**. The eluant was 10% MeOH in DCM with 5% aq. NH_3 (23%). This was further purified by preparative HPLC equipped with a

Luna 5 µm C-18 (2) 100 Å column (250 mm × 21.2 mm). HPLC Method: Mobile phase: linear gradient solvent system, 10 mM TEAA (triethylammonium acetate)-CH_3CN from 85:15 to 50:50 in 40 min; Retention time: ~29.2 min; Compound (3.0 mg, 39%) was isolated as a white solid with traces of TEAA buffer.

^{1}H NMR (400 MHz, methanol-*d*$_4$) δ 8.59 (s, 1H), 7.46 (s, 1H), 6.23 (d, *J* = 5.2 Hz, 1H), 4.45 (t, *J* = 5.1 Hz, 1H), 4.29 (t, *J* = 4.7 Hz, 1H), 4.11 (q, *J* = 3.4 Hz, 1H), 3.86 (dd, *J* = 12.0, 3.0 Hz, 1H), 3.76 (dd, *J* = 12.2, 3.1 Hz, 1H), 2.54 (s, 3H).

HRMS (ESI) m/z: $[M+H]^+$ calculated for $C_{12}H_{17}N_4O_4$ 281.1250; found 281.1253.

Purity by analytical HPLC: > 98%. Rt: 5.01 min.

Sample in DMSO-*d*$_6$ was used for 1D-NOE, and 2D-NMR.

^{1}H NMR (500 MHz, DMSO-*d*$_6$) δ 8.41 (s, 1H), 7.43 (s, 1H), 6.03 (d, *J* = 5.4 Hz, 1H), 5.96 (s, 2H), 5.56 (d, *J* = 5.9 Hz, 1H), 5.30 – 5.21 (m, 1H), 5.10 (d, *J* = 5.5 Hz, 1H), 4.33 (q, *J* = 5.1 Hz, 1H), 4.10 (q, *J* = 4.0 Hz, 1H), 3.95 (q, *J* = 3.8 Hz, 1H), 3.66 (dt, *J* = 12.2, 4.1 Hz, 1H), 3.57 (dt, *J* = 12.1, 3.9 Hz, 1H), 2.42 (s, 3H).

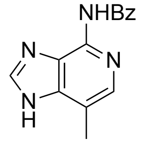

12

***N*-(7-Methyl-1*H*-imidazo[4,5-c]pyridin-4-yl)benzamide (12):** To a stirred suspension of compound **5** (200 mg; 1.350 mmol, 1.0 eq.) in dry pyridine (6 mL), chlorotrimethylsilane (1.03 mL, 8.108 mmol, 6.0 eq.) was added drop wise at 0ºC and the cooled mixture was stirred for 1 h. Benzoyl chloride (0.942 mL, 8.108 mmol, 6.0 eq.) was added drop wisely

via syringe and the reaction mixture was stirred for 1 h with cooling and overnight at room temperature. The mixture was quenched by the addition of ice flakes at 0ºC, and stirred for 15 min. After addition of aqueous ammonia solution (4.0 ml, 23%), the reaction mixture was stirred for 3 h at room temperature. The solvent was removed using a rotary evaporator and co-evaporated with toluene (2 x 5 ml). The crude product was purified by column chromatography on SiO_2. Yield: 730 mg of **12** as white solid (43 %). The eluant was 20-30% acetone in hexane.

^{1}H NMR (400 MHz, a few drops of CD_3OD in chloroform-*d*) δ 8.10 (dd, *J* = 4.5, 1.8 Hz, 1H), 7.99 – 7.90 (m, 2H), 7.78 (d, *J* = 4.5 Hz, 1H), 7.51 (tq, *J* = 6.6, 1.5 Hz, 1H), 7.42 (dd, *J* = 7.1, 1.5 Hz, 1H), 7.32 – 7.22 (m, 1H)2.48 (d, *J* = 2.6 Hz, 3H)., 2.47 (s, 2H).
HRMS (ESI) m/z: $[M+H]^+$ calculated for $C_{14}H_{13}N_4O$ 253.1089; found 253.1087.

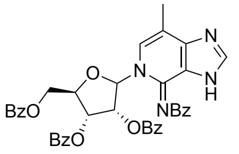

13

(3*S*,4*S*,5*S*)-2-((*Z*)-4-(Benzoylimino)-7-methyl-3,4-dihydro-5*H*-imidazo[4,5-*c*]pyridin-5-yl)-5-((benzoyloxy)methyl)tetrahydrofuran-3,4-diyl dibenzoate (13): Bis(trimethylsilyl)acetamide (BSA, 1.02 mL, 4.164, 3.0 eq.) was added to a stirred solution of **12** (350 mg, 1.388 mmol, 1.0 eq.) and 1-*O*-acetyl-2,3,5-tri-*O*-benzoyl-D-ribofuranose **8**

(700 mg, 1.39 mmol, 1.0 eq.) in dried acetonitrile (12 mL) and stirring was continued under N_2 at 50 °C for 20 min. To this clear solution, TMSOTf (0.28 mL, 1.527mmol, 1.1 eq.) was added and the reaction mixture stirred under N_2 at 80 °C for 16 h. The mixture was cooled to rt, and the volatiles were removed using a rotary evaporator to obtain an oily residue. The residue was dissolved in EtOAc (50 mL), washed with saturated $NaHCO_3$ (20 mL), and brine (20 mL), dried over anhydrous Na_2SO_4, filtered, and concentrated using a rotary evaporator to obtain the crude product. The crude product was purified by column chromatography to give the protected nucleoside **13** (683 mg, 71%) as a white solid and the bis-adduct **14** as a white solid (79 mg, 5%). The eluant was 10-20% acetone in DCM.

^{1}H NMR (600 MHz, DMSO-*d*$_6$) δ 13.12 (s, 1H), 8.45 (s, 1H), 8.22 (d, *J* = 7.7 Hz, 3H), 8.08 – 7.99 (m, 3H), 7.96 (d, *J* = 7.7 Hz, 2H), 7.86 – 7.62 (m, 6H), 7.56 (t, *J* = 7.7 Hz, 2H), 7.45 (dt, *J* = 14.5, 7.6 Hz, 3H), 7.18 (t, *J* = 7.6 Hz, 2H), 6.10 (t, *J* = 2.8 Hz, 2H), 5.15 (q, *J* = 4.3 Hz, 1H), 5.05 (dd, *J* = 12.7, 3.1 Hz, 1H), 4.95 (dd, *J* = 12.6, 4.2 Hz, 1H), 2.33 (s, 3H). HRMS (ESI) m/z: [M+H]$^+$ calculated for $C_{40}H_{33}N_4O_8$ 697.2298; found 697.2303.

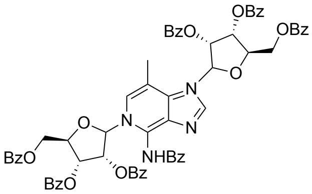

14

(3*S*,3'*S*,4*S*,4'*S*,5*S*,5'*S*)-(4-Benzamido-7-methyl-5*H*-1λ^4-imidazo[4,5-*c*]pyridine-1,5-diyl)bis(5-((benzoyloxy)methyl)tetrahydrofuran-2,3,4-triyl) tetrabenzoate (14):

^{1}H NMR (400 MHz, chloroform-*d*) δ 8.14 (t, *J* = 7.2 Hz, 4H), 8.07 (s, 1H), 8.03 (d, *J* = 7.7 Hz, 2H), 7.95 – 7.86 (m, 8H), 7.67 – 7.35 (m, 17H), 7.28 (ddt, *J* = 14.9, 8.6, 5.1 Hz, 7H),

7.19 (t, *J* = 7.6 Hz, 3H), 6.44 (d, *J* = 6.1 Hz, 1H), 6.07 (t, *J* = 5.8 Hz, 1H), 5.92 – 5.86 (m, 3H), 5.01 – 4.89 (m, 1H), 4.85 – 4.68 (m, 5H), 4.64 – 4.57 (m, 1H), 2.15 (s, 3H).

HRMS (ESI) m/z: $[M+H]^+$ calculated for $C_{66}H_{53}N_4O_{15}$ 1141.3507; found 1141.3525.

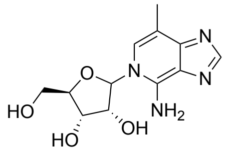

15

(3*S*,4*R*,5*S*)-2-(4-Amino-7-methyl-5*H*-imidazo[4,5-*c*]pyridin-5-yl)-5-(hydroxymethyl)tetrahydrofuran-3,4-diol (15): In a 25 mL sealed tube were added compound (**13**) (120 mg, 0.312 mmol) and saturated ammonia in MeOH (5.0 mL). The mixture was sealed and the reaction mixture stirred at 80 °C for 16 h. The solvent was removed and co-evaporated with ethanol. The crude product was purified by column chromatography to give the acetate salt of derivative **15** (72 mg, 83%) as a white solid. The eluant was 15-20% MeOH in DCM.

^{1}H NMR (600 MHz, DMSO-d_6) δ 7.84 (d, *J* = 0.9 Hz, 1H), 7.51 – 7.45 (m, 1H), 5.86 (d, *J* = 6.0 Hz, 1H), 4.25 (t, *J* = 5.7 Hz, 1H), 4.09 (dd, *J* = 5.4, 3.4 Hz, 1H), 4.04 (q, *J* = 3.0 Hz, 1H), 3.73 (dd, *J* = 12.0, 2.9 Hz, 1H), 3.68 (dd, *J* = 12.1, 2.8 Hz, 1H), 2.26 (d, *J* = 1.1 Hz, 3H).

HRMS (ESI) m/z: $[M+H]^+$ calculated for $C_{12}H_{17}N_4O_4$ 281.1250; found 281.1250.

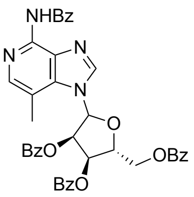

16

(3*R*,4*R*,5*R*)-2-(4-Benzamido-7-methyl-1*H*-imidazo[4,5-*c*]pyridin-1-yl)-5-((benzoyloxy)methyl)tetrahydrofuran-3,4-diyl dibenzoate (16): Bis(trimethylsilyl)acetamide (BSA, 2.10 mL, 8.56 mmol, 3.0 eq.) was added to a stirred solution of **12** (0.720 g, 2.854 mmol, 1.0 eq.) and 1-*O*-acetyl-2,3,5-tri-*O*-benzoyl-D-ribofuranose (**8**, 1.58 g, 3.14 mmol, 1.1 eq.) in dry toluene (25 mL) and stirring was continued under N_2 at 50 °C for 20 min. To this clear solution, TMSOTf (1.56 mL, 8.562 mmol, 3.0 mmol) was added and the reaction mixture stirred under N_2 at gentle reflux for 16 h. The mixture was cooled to rt, and the volatiles were removed using a rotary evaporator to obtain an oily residue. The residue was dissolved in DCM (100 mL), washed with saturated $NaHCO_3$ (20 mL), and brine (20 mL), dried over anhydrous Na_2SO_4, filtered, and the solvent was removed using a rotary evaporator. The crude product was purified by column chromatography to give the protected nucleoside **16** (547 mg, 27%) as a white solid. The eluant was 5-15% acetone in DCM.

^{1}H NMR (600 MHz, DMSO-d_6) δ 10.70 (s, 1H), 8.77 (s, 1H), 8.06 -8.04 (m, 3H), 7.97 – 7.91 (m, 6H), 7.68 – 7.65 (m, 3H), 7.62 – 7.60 (m, 1H), 7.55 -7.50 (m, 5H), 7.49 – 7.45 (m, 4H), 6.82 (d, *J* = 5.4 Hz, 1H), 6.31 (t, *J* = 5.8 Hz, 1H), 6.03 (q, *J* = 4.6, 3.5 Hz, 1H), 4.92 (q, *J* = 4.7 Hz, 1H), 4.78 – 4.70 (m, 2H), 2.68 (s, 3H).

HRMS (ESI) m/z: $[M+H]^+$ calculated for $C_{40}H_{33}N_4O_8$ 697.2298; found 697.2303.

Preparation of compound 3 from 16: In a 25 mL sealed tube, saturated ammonia in MeOH (5.0 mL) and compound **16** (56 mg, 0.0804 mmol) were taken and the solution was stirred at 80 ^{o}C for three days. Volatiles were evaporated using a rotary evaporator, and the crude product was purified by column chromatography to give **(3)** (13 mg, 57%) as a white solid.

Analytical spectra and structural assignment of synthesized compounds

^{1}H NMR (400 MHz, $CDCl_3$) of compound 9.

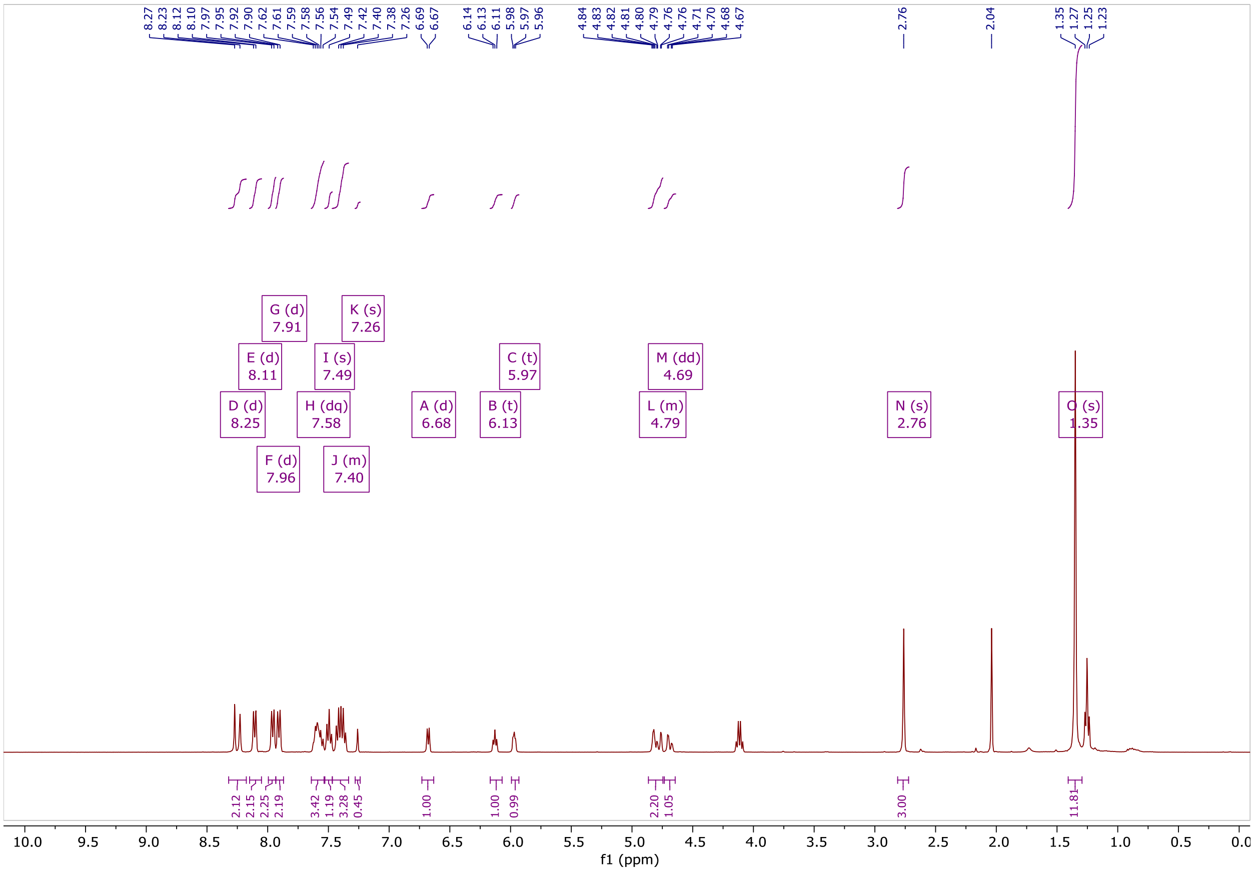

^{1}H NMR (400 MHz, $CDCl_3$) of compound 10.

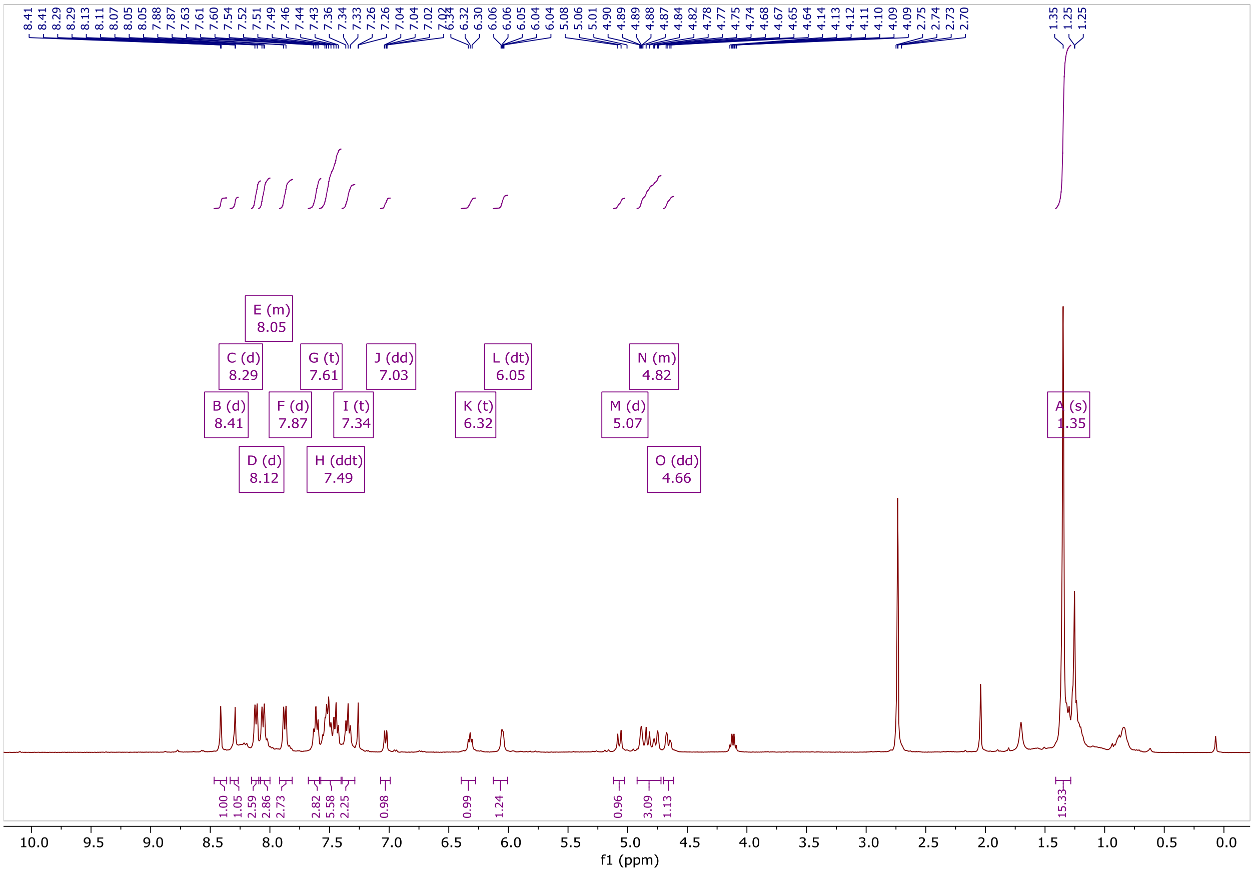

^{1}H NMR (400 MHz, $CDCl_3$) of compound 11.

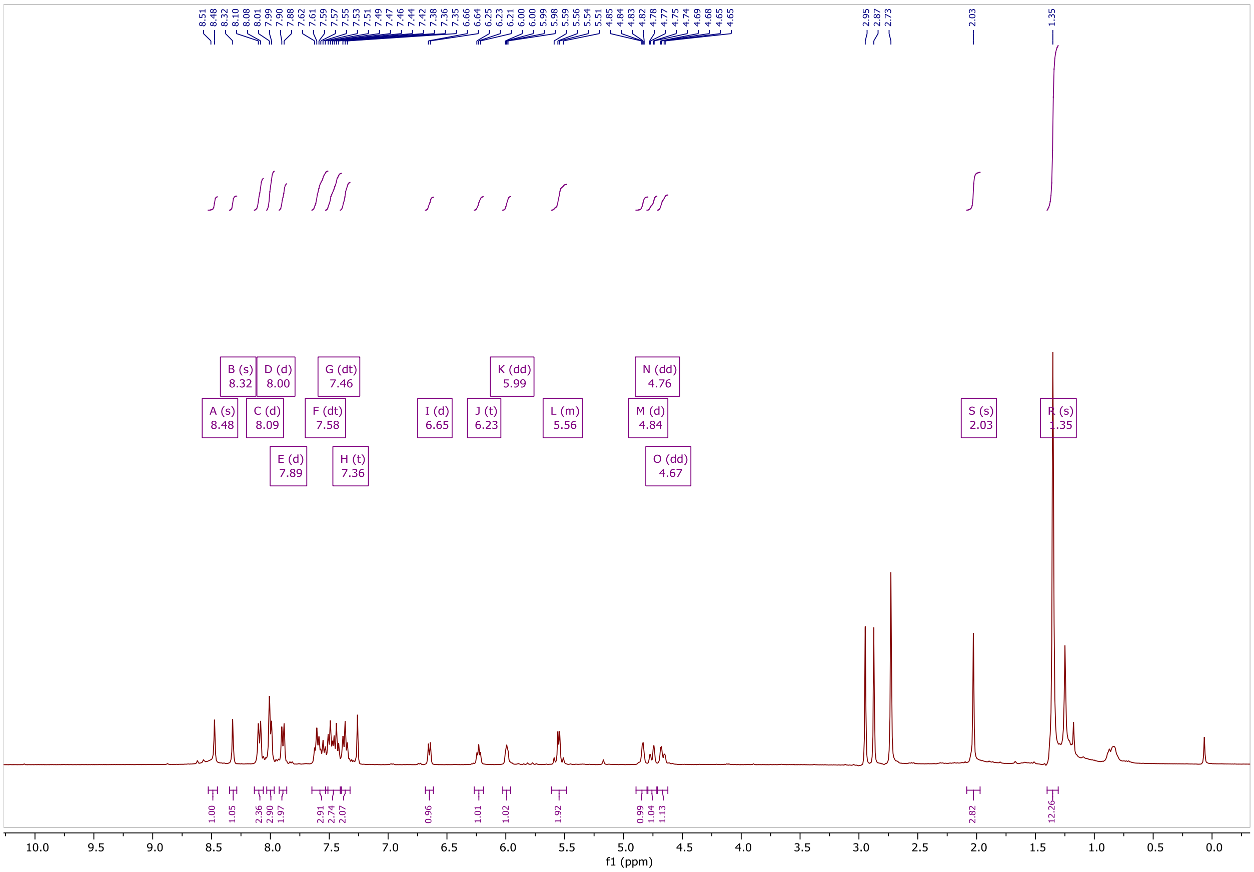

^{1}H NMR (400 MHz, CD_3OD) of compound 4.

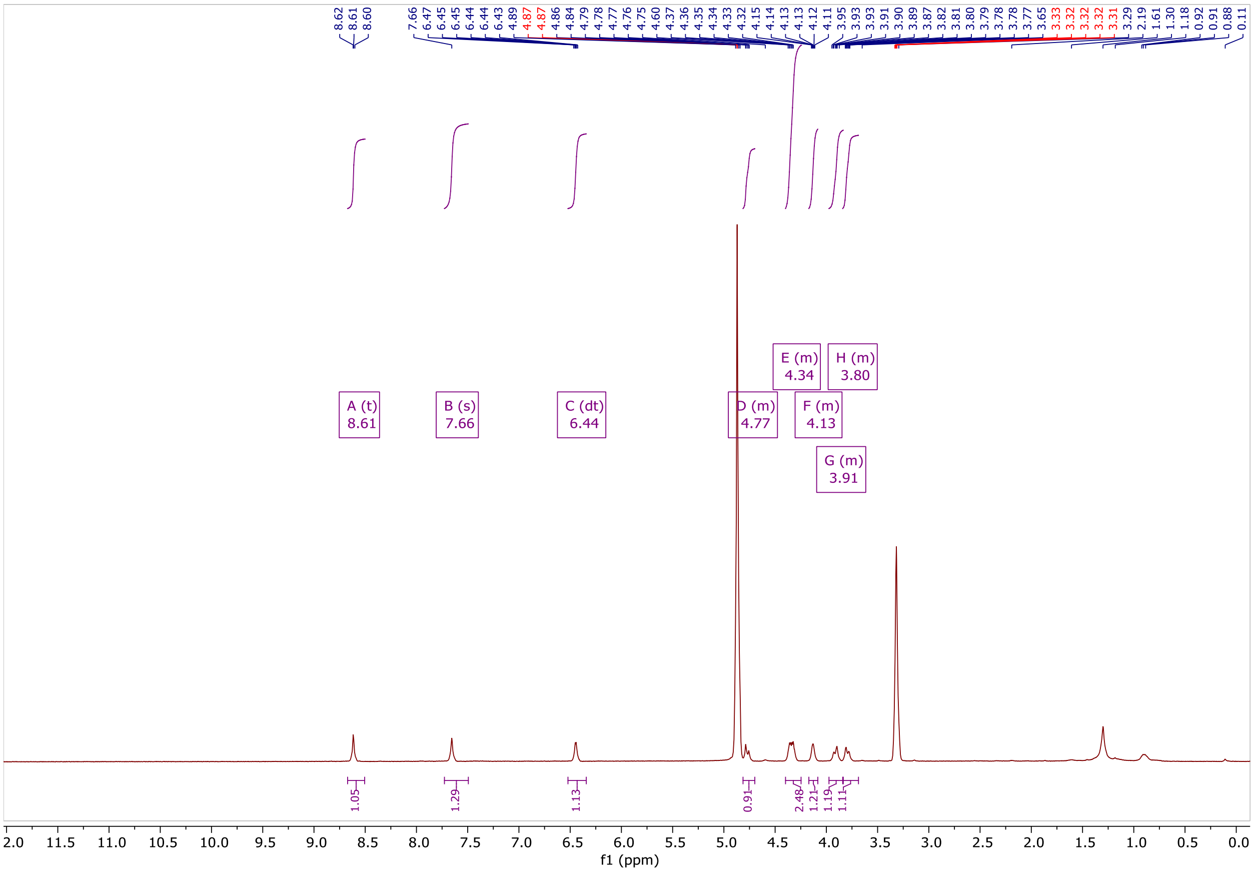

HR-MS for compound 4.

Elemental Composition Report **Page 1**

Single Mass Analysis
Tolerance = 10.0 mDa / DBE: min = -2.0, max = 1000.0
Element prediction: Off
Number of isotope peaks used for i-FIT = 3

Monoisotopic Mass, Even Electron Ions
44 formula(e) evaluated with 2 results within limits (up to 19 closest results for each mass)
Elements Used:
C: 0-100 H: 0-200 N: 4-4 O: 0-20
31-Oct-2017
ram-31oct17-119-ii 86 (1.590) Cn (Cen,3, 50.00, Ar); Sm (SG, 3x5.00); Sb (12,5.00) TOF MS ES+
5.82e+003

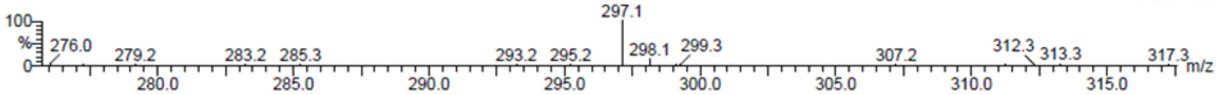

	Mass	Calc. Mass	mDa	PPM	DBE	i-FIT	Formula
Minimum:					-2.0		
Maximum:			10.0	10.0	1000.0		
	297.1201	297.1199	0.2	0.7	6.5	9.6	C12 H17 N4 O5
		297.1140	6.1	20.5	15.5	126.4	C19 H13 N4

Analytical HPLC chromatogram for compound 4.

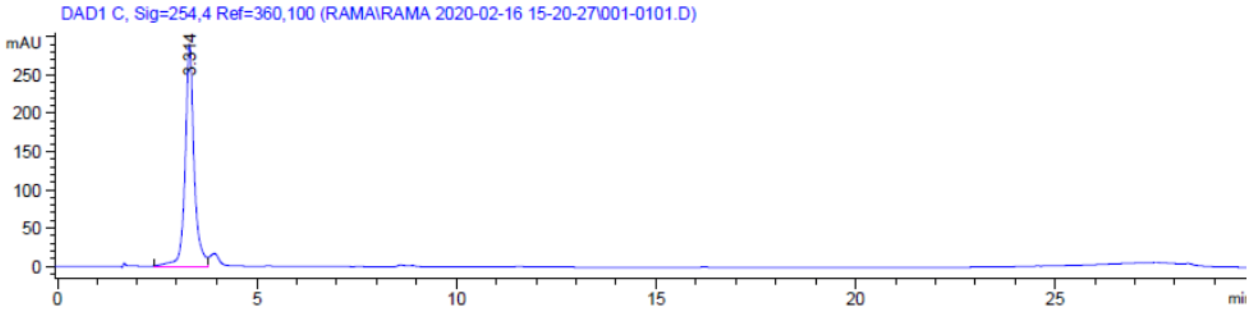

^{1}H NMR (400 MHz, CD_3OD) of compound 3.

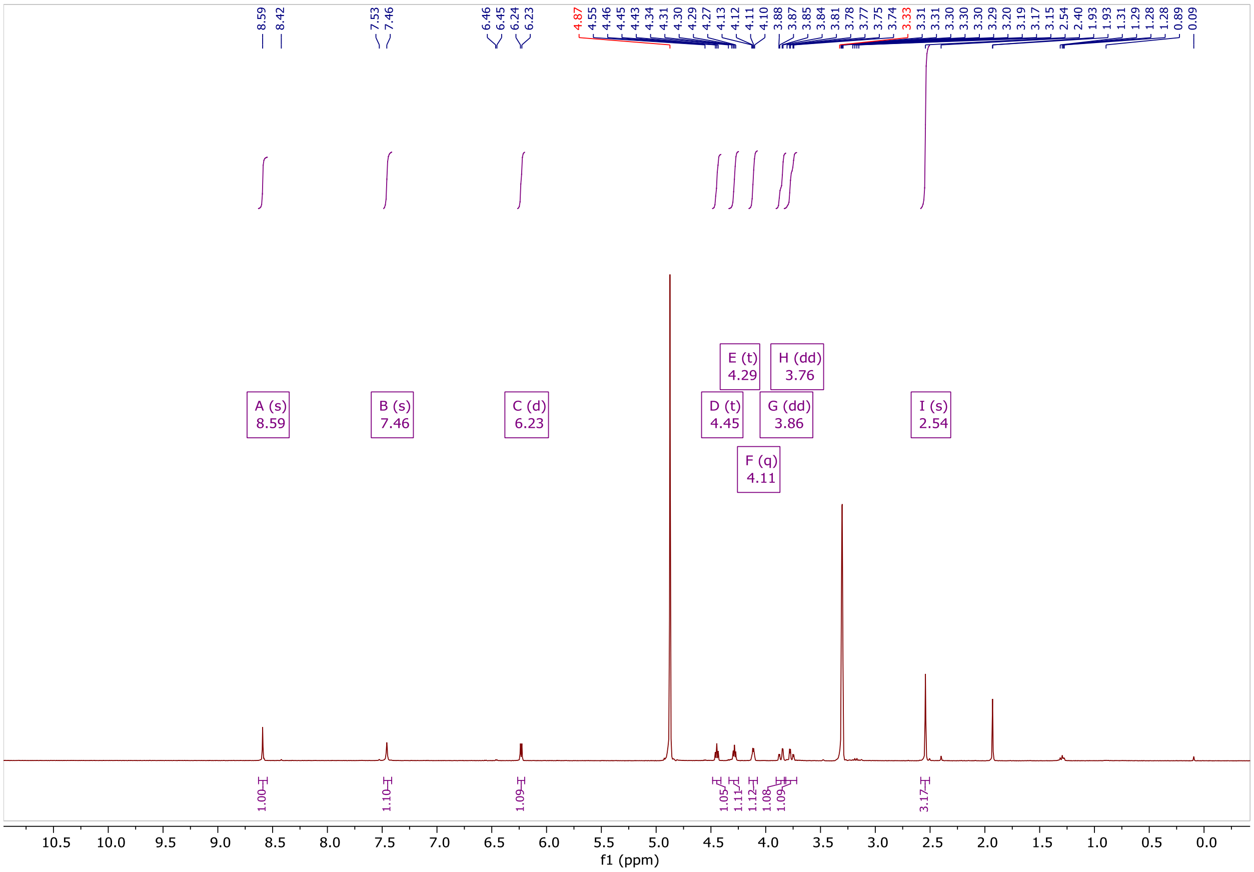

HR-MS for compound 3.

Elemental Composition Report **Page 1**

Single Mass Analysis
Tolerance = 3.0 mDa / DBE: min = -1.5, max = 100.0
Element prediction: Off
Number of isotope peaks used for i-FIT = 3

Monoisotopic Mass, Even Electron Ions
38 formula(e) evaluated with 1 results within limits (up to 50 closest results for each mass)
Elements Used:
C: 0-75 H: 0-200 N: 4-4 O: 0-25
RAM-30NOV18-289-CC 110 (1.878) AM2 (Ar,25000.0,0.00,0.00); ABS
TOF MS ES+

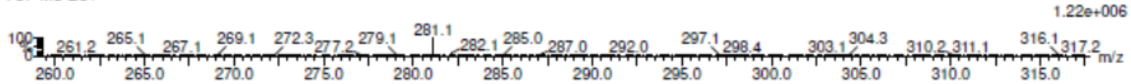

Minimum:				-1.5				
Maximum:		3.0	5.0	100.0				
Mass	Calc. Mass	mDa	PPM	DBE	i-FIT	Norm	Conf(%)	Formula
281.1253	281.1250	0.3	1.1	6.5	355.3	n/a	n/a	C12 H17 N4 O4

Analytical HPLC chromatogram for compound 3:

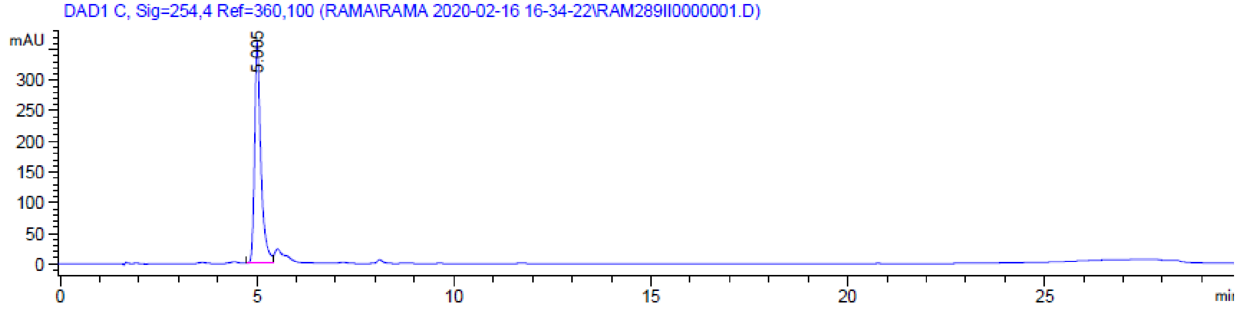

^{1}H NMR (500 MHz, $(CD_3)_2SO$) of compound 3.

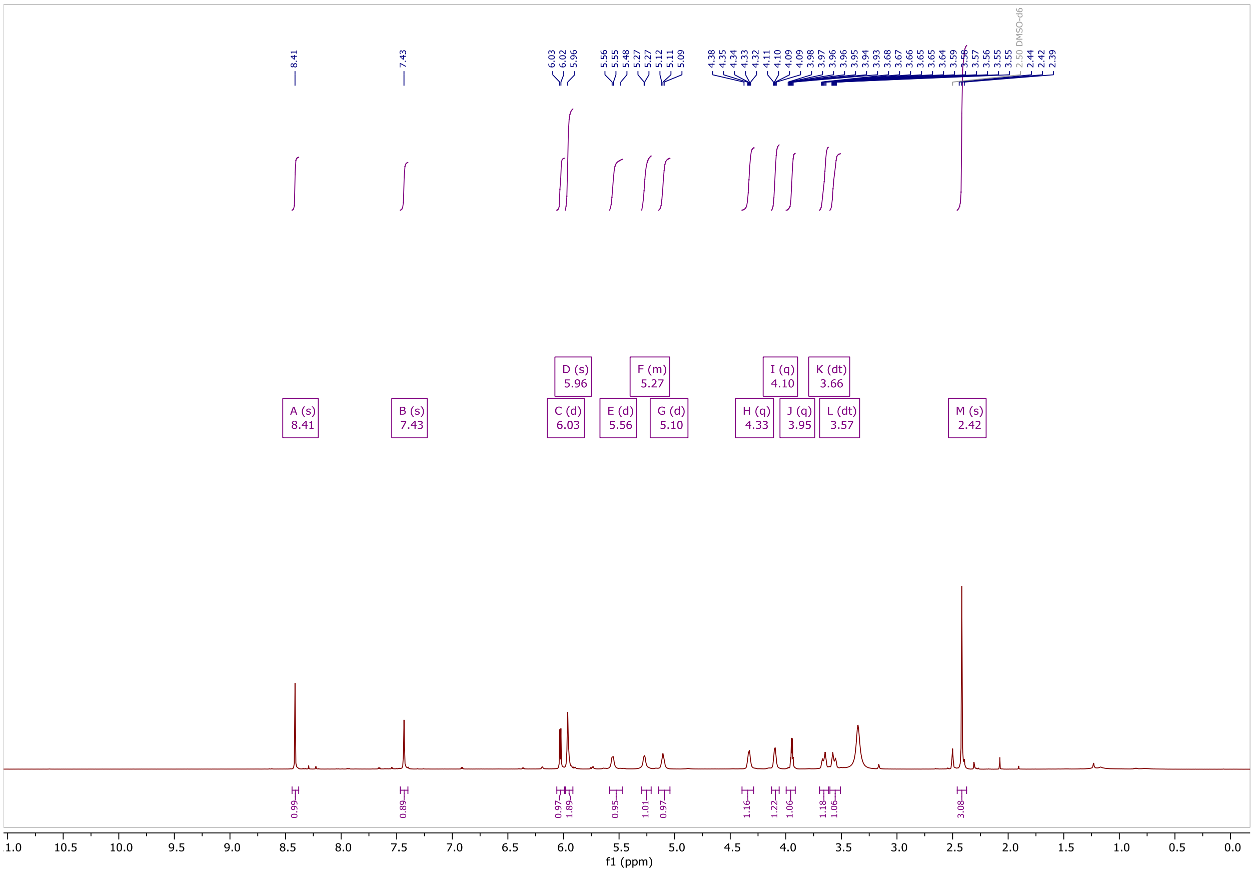

^{1}H NMR (400 MHz, a few drops of CD_3OD in $CDCl_3$) of compound 12.

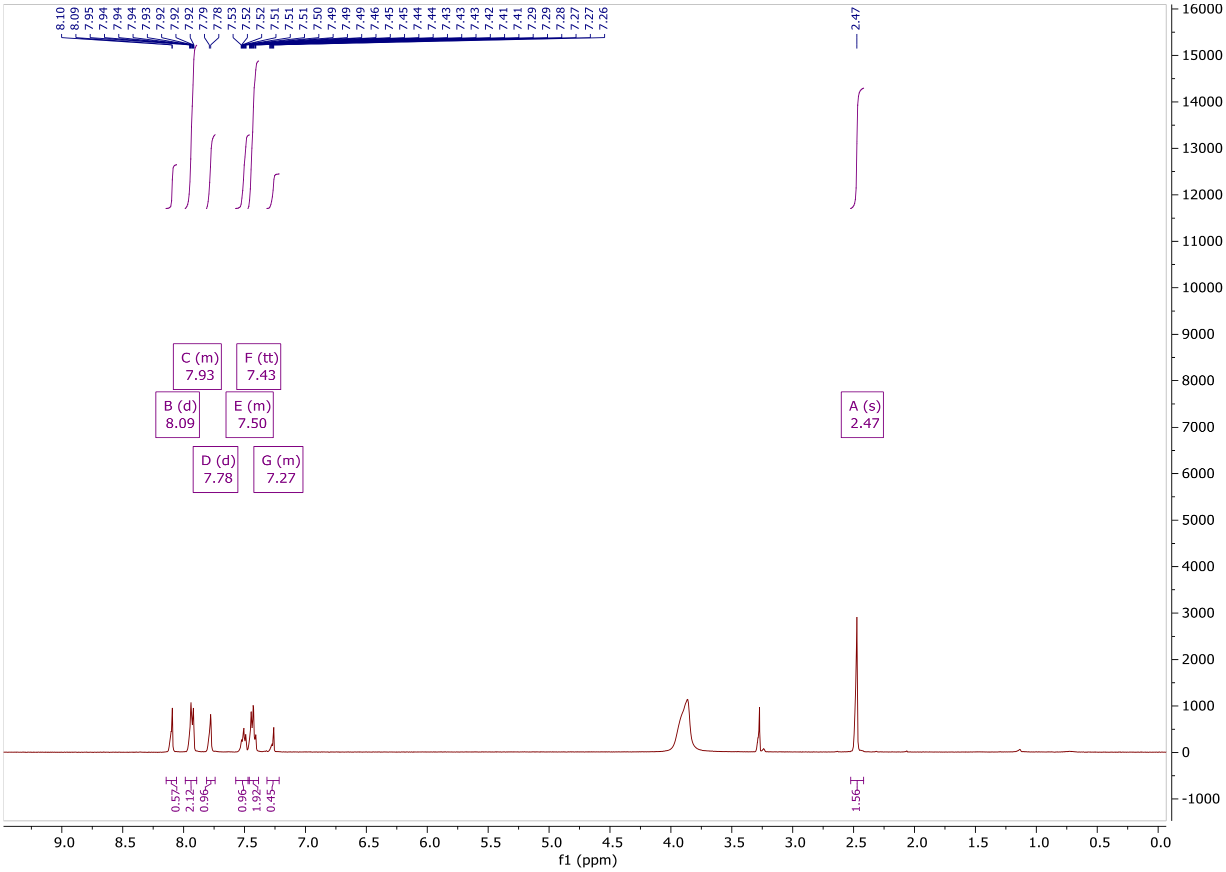

^{1}H NMR (600 MHz, $(CD_3)_2SO$) of compound 13.

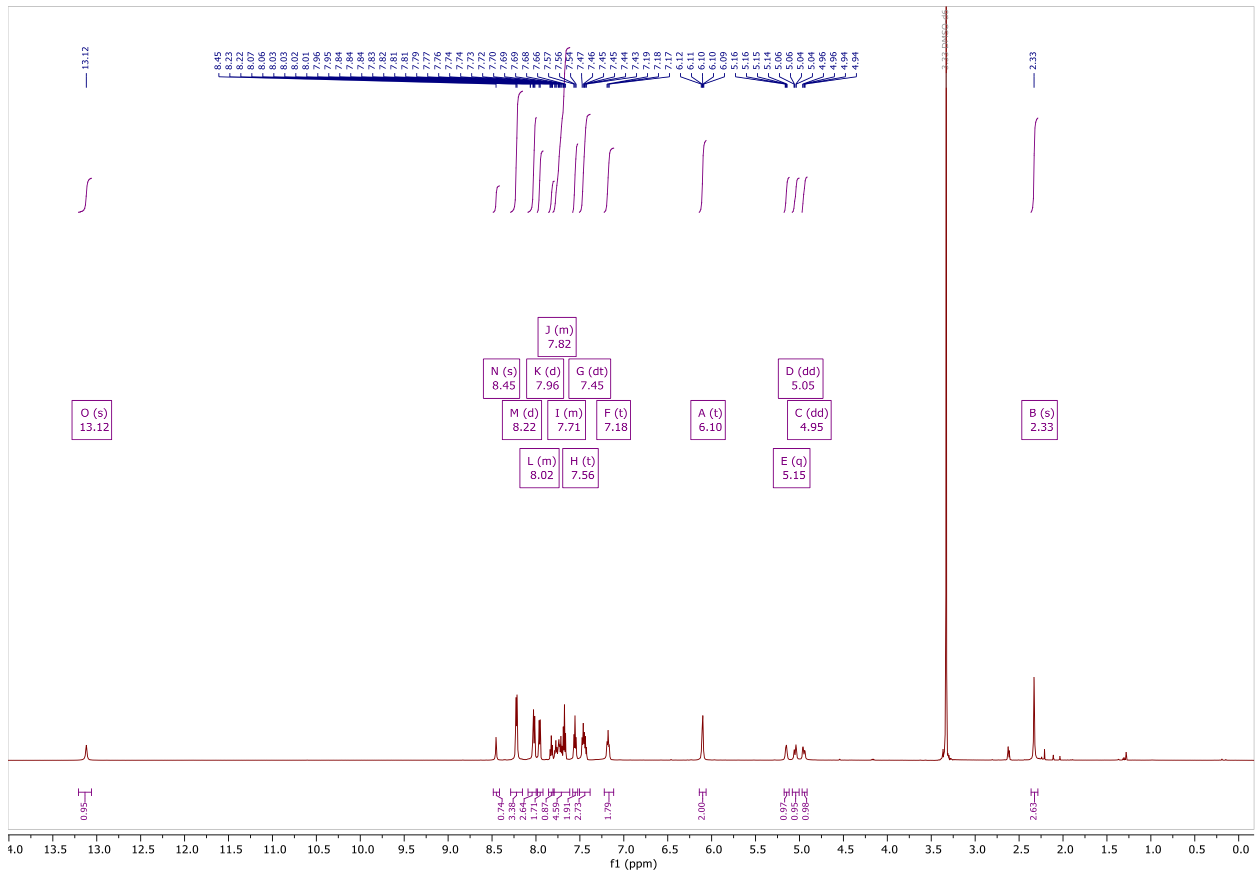

^{1}H NMR (500 MHz, $CDCl_3$) of compound 14.

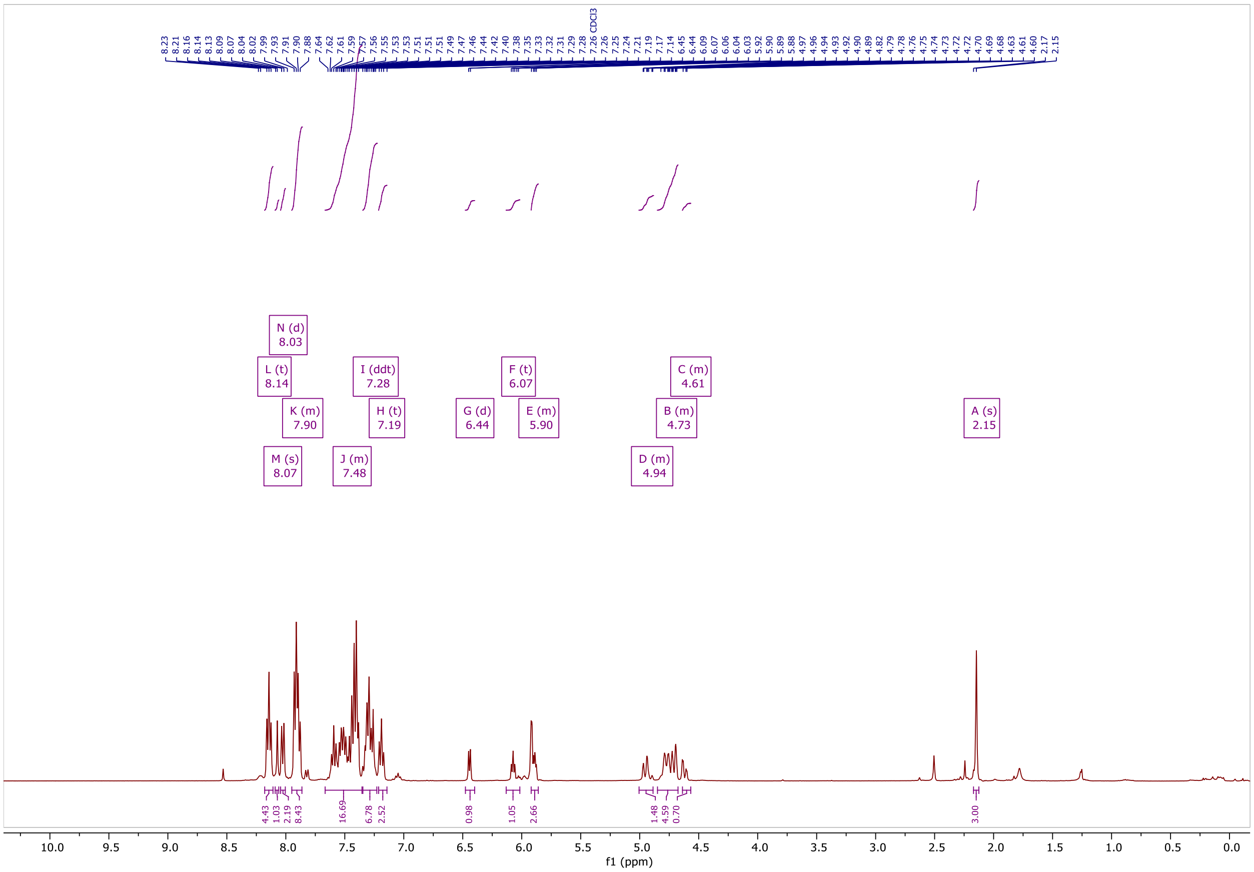

^{1}H NMR (600 MHz, $(CD_3)_2SO$) of compound 15.

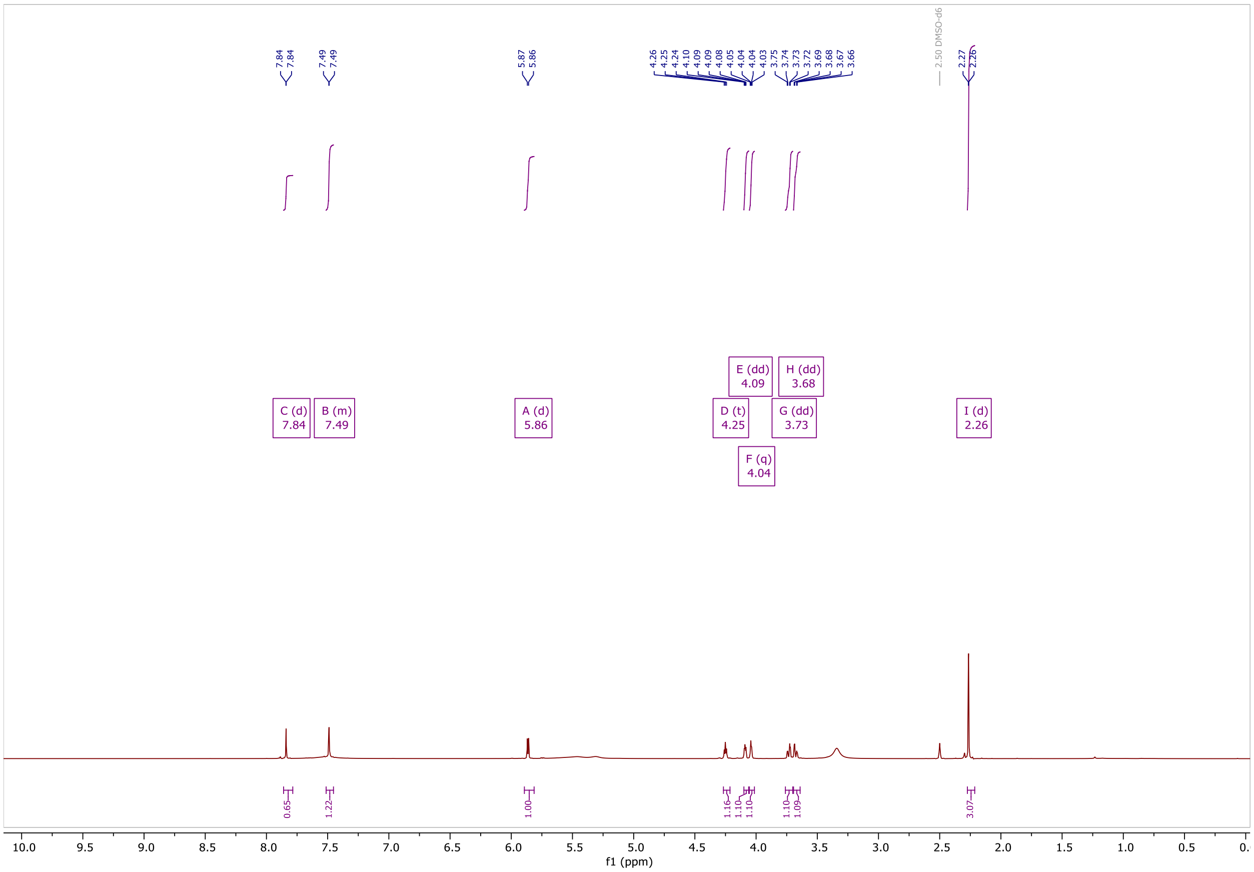

^{1}H NMR (600 MHz, $(CD_3)_2SO$) of compound 16.

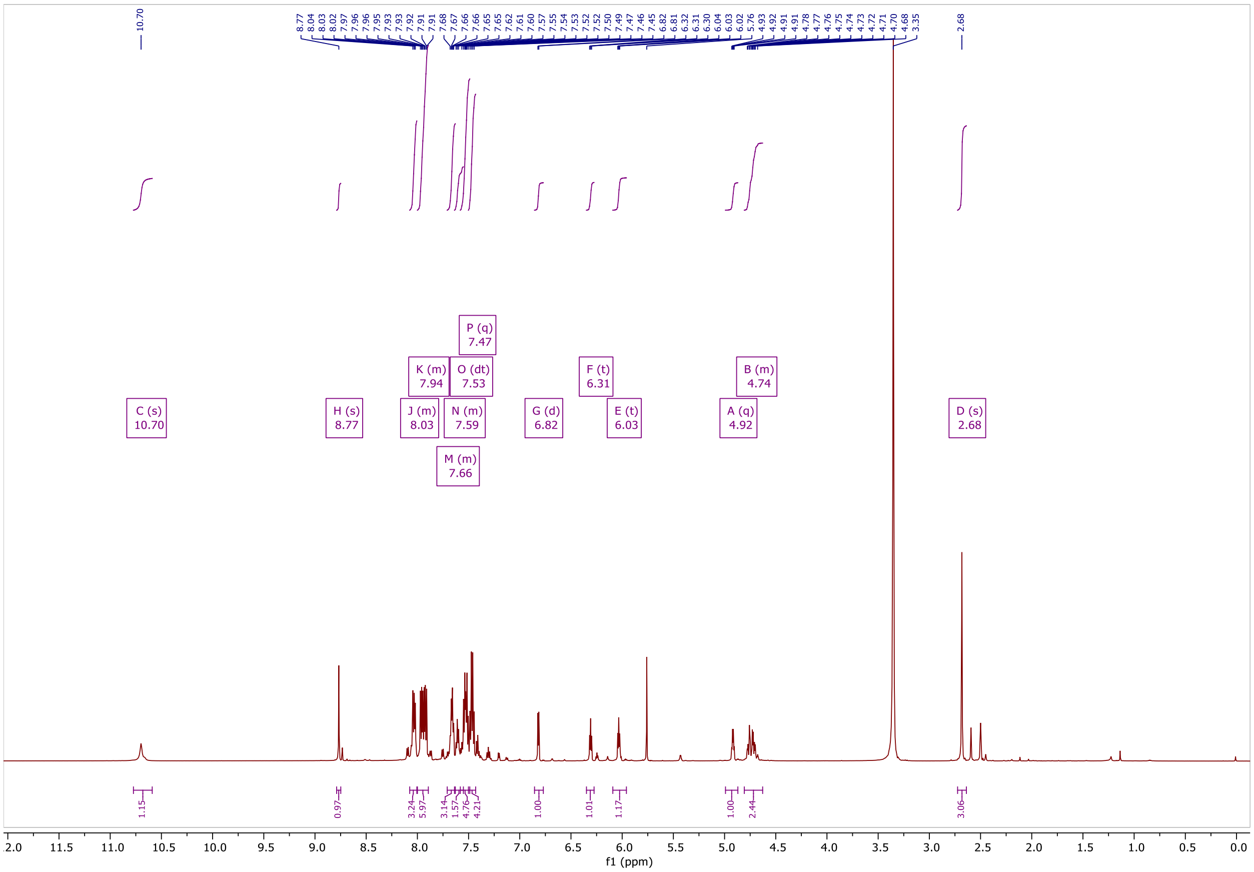

Supplementary Analytical Figures

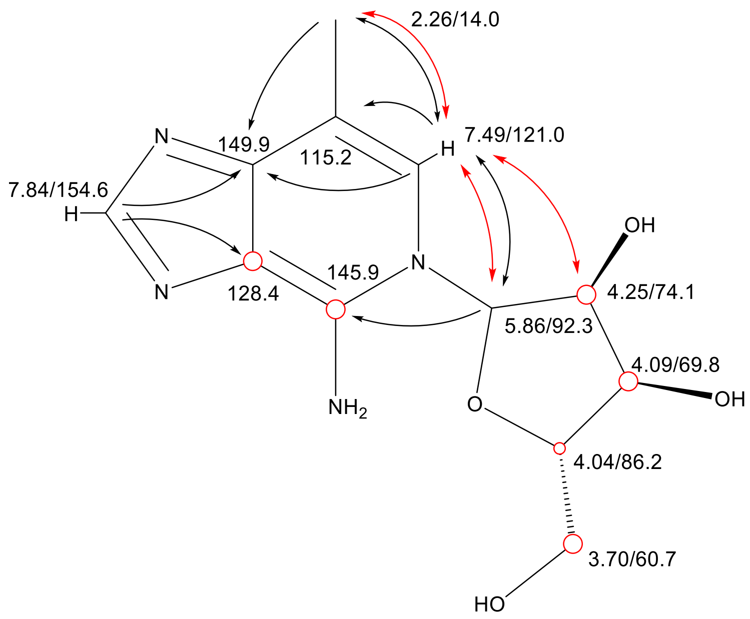

Figure S2. Scheme S2, #15 resonance assignments ($^{1}H/^{13}C$ ppm) in DMSO-d_6 at 298K and pertinent NOE, HMBC, and isotope shift data. Larger (~10 Hz) isotope shifts are represented by larger circles.

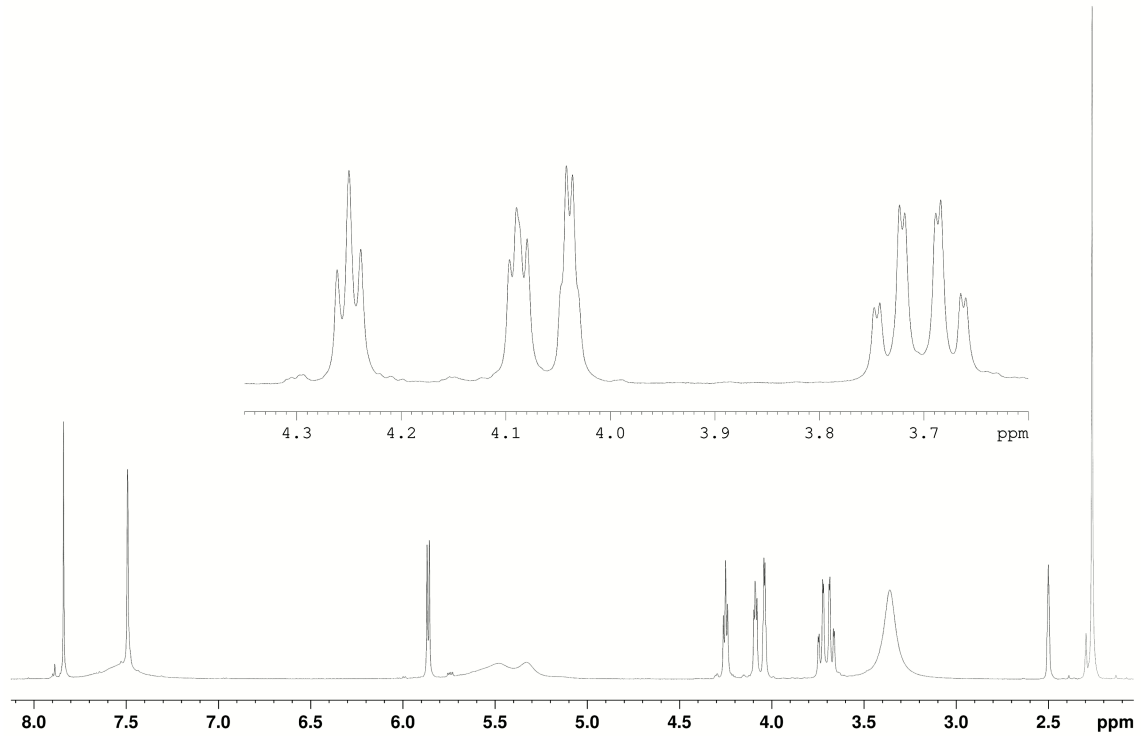

Figure S3. ^{1}H spectrum of Scheme S2, #15 in DMSO-d_6 at 298K. Assignments are in Figure S2.

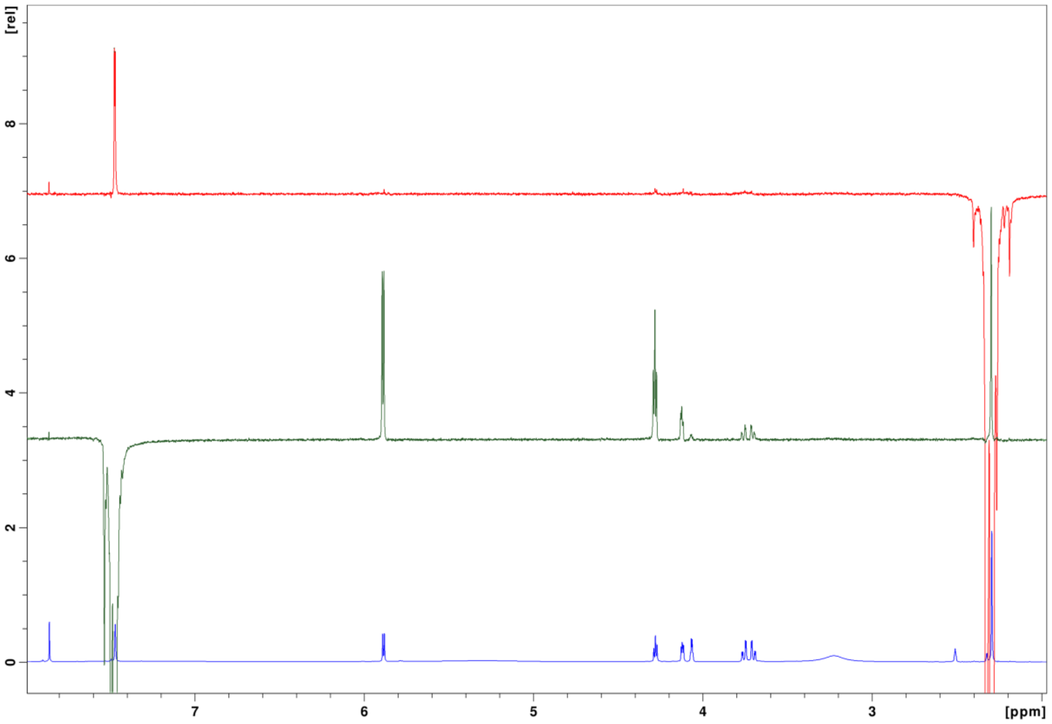

Figure S4. 1D-dpfgse spectra of Scheme S2, #15 at 328K. The blue spectrum is for reference. The green dpfgse spectrum selectively inverts only the ^{1}H-7.49 ppm resonance, with nearby ^{1}Hs positive. The red dpfgse spectrum shows the 7.49 resonance is near the methyl (inverted). There were no NOE responses from the 7.84 resonance (not shown).

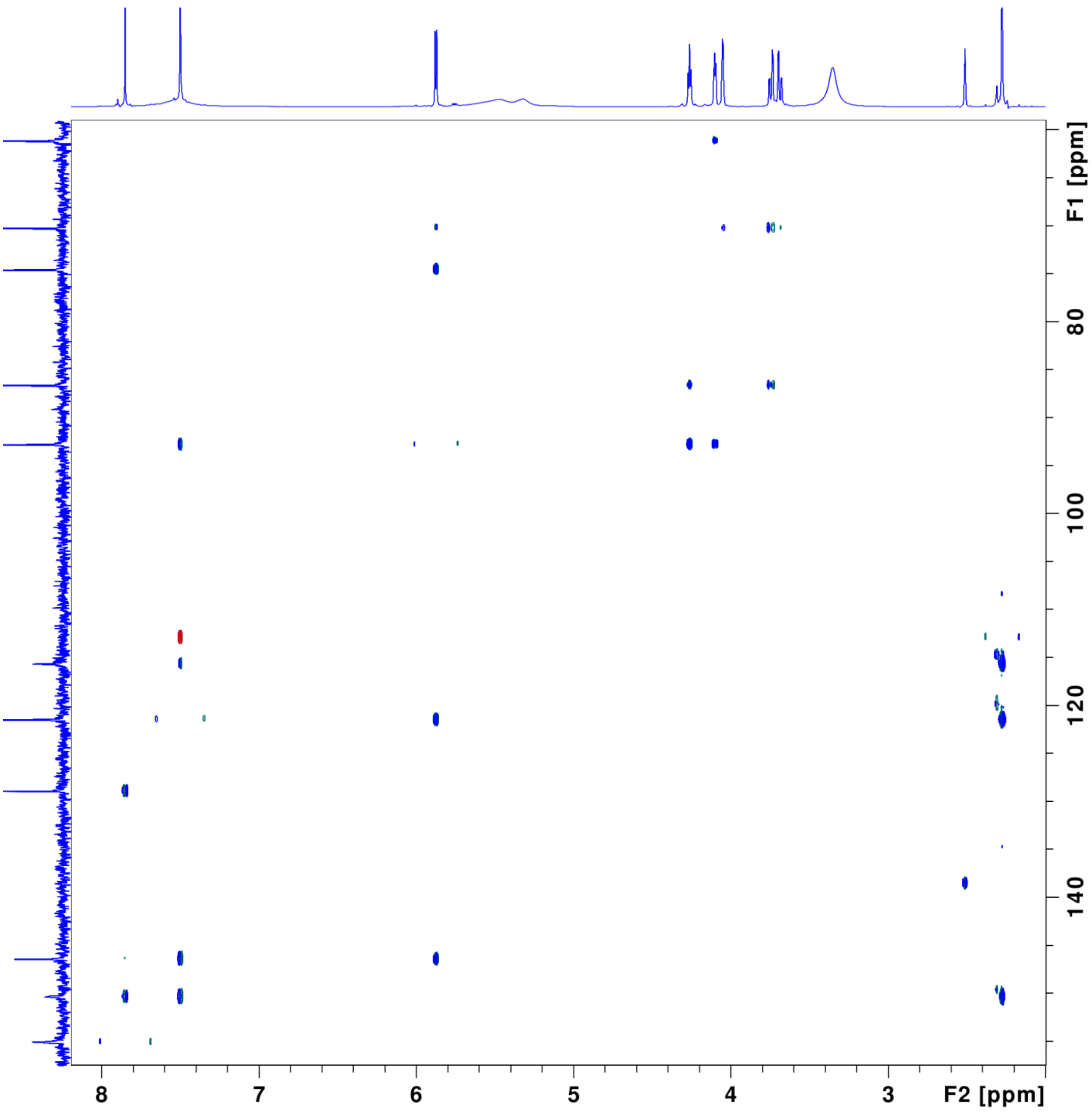

Figure S5. HMBC spectrum of Scheme S2, #15 at 298K optimized for an 8 Hz coupling. The red correlation is aliased from the methyl at 14 ppm.

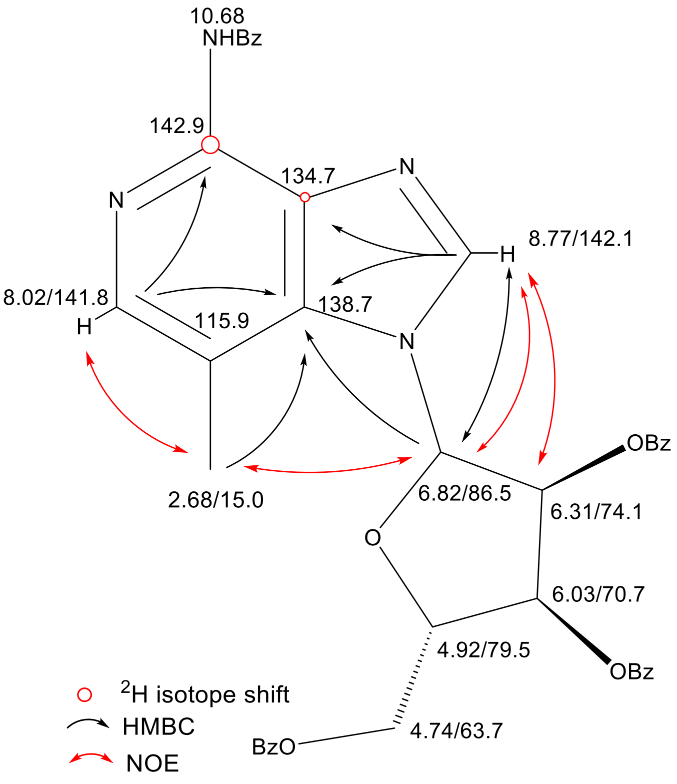

Figure S6. Scheme S2, #16 resonance assignments ($^{1}H/^{13}C$ ppm) in DMSO-d_6 at 298K and pertinent NOE, HMBC, and isotope shift data. Larger (~10 Hz) isotope shifts are represented by larger circles.

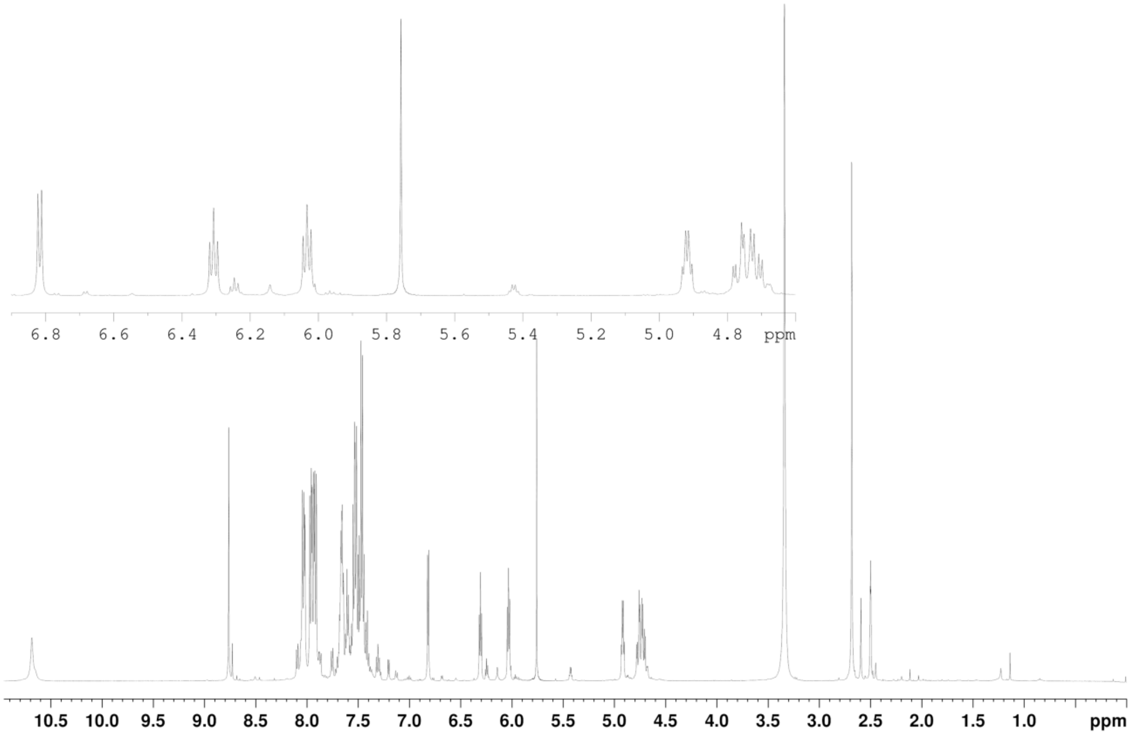

Figure S7. ^{1}H spectrum of Scheme S2, #16 in DMSO-d_6 at 298K. Assignments are in Figure S6.

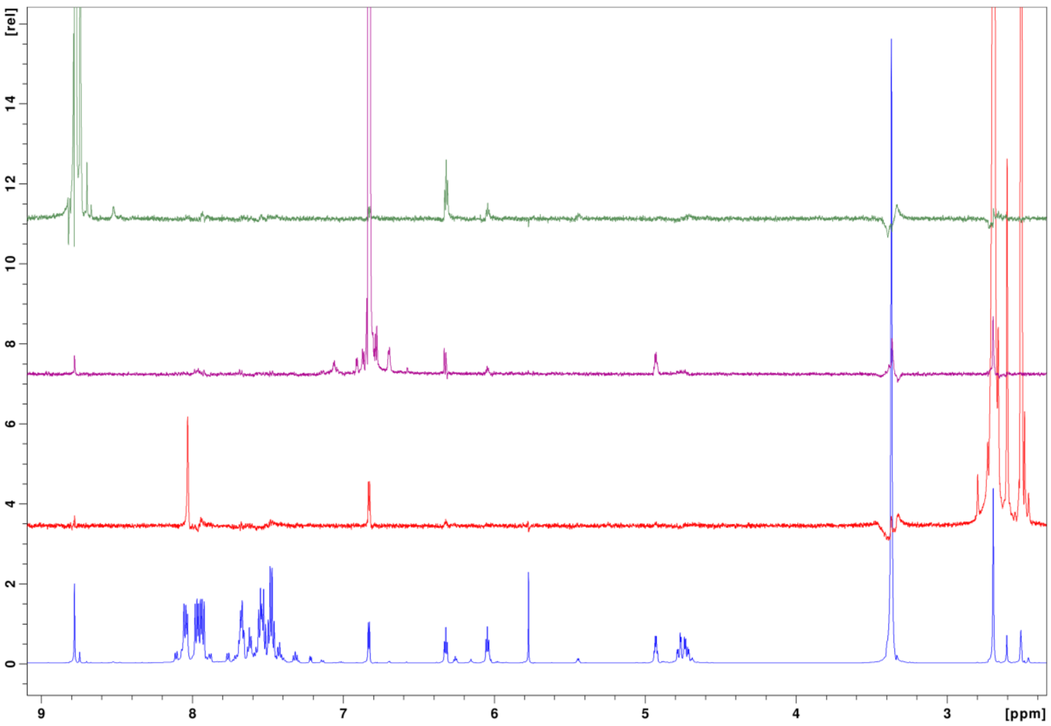

Figure S8. 1D-dpfgse spectra of Scheme S2, #16 at 294K, which is in the slow tumbling regime. The blue spectrum is for reference. The green dpfgse spectrum selectively inverts the 8.77 ppm ^{1}H and shows that the ribose 6.3 ppm ^{1}H is nearest. The purple spectrum selects the 6.8 ppm ribose ^{1}H, showing that the ribose 4.9 and 6.3 ppm ^{1}Hs as well as the 8.77 purine ^{1}H are near it. The red dpfgse spectrum shows the ^{1}Hs near the methyl.

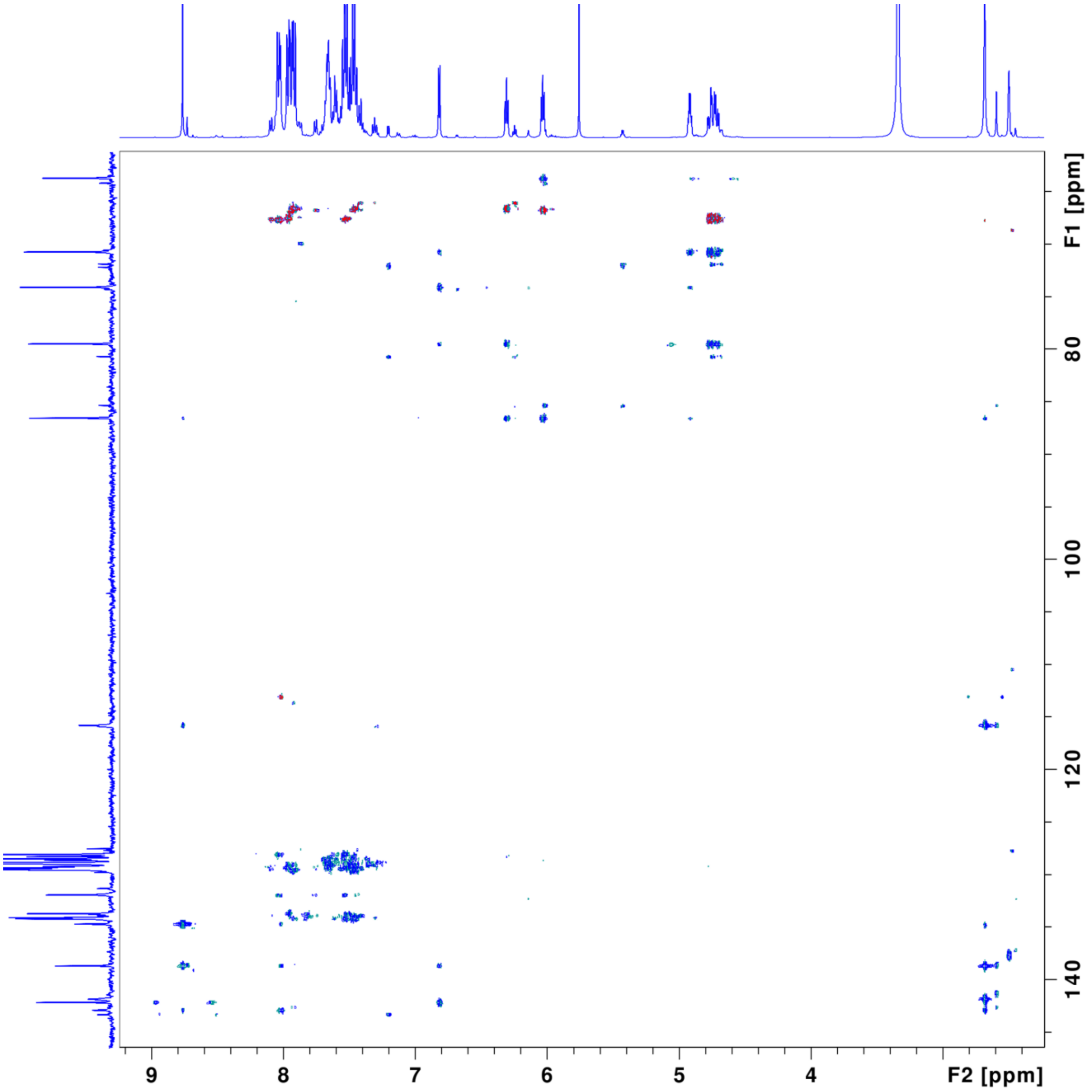

Figure S9. HMBC spectrum of Scheme S2, #16 at 298K optimized for a 4 Hz coupling. The red correlations are aliased, the one at 113 ppm from the methyl and those from 67-69 ppm from the carbonyls.

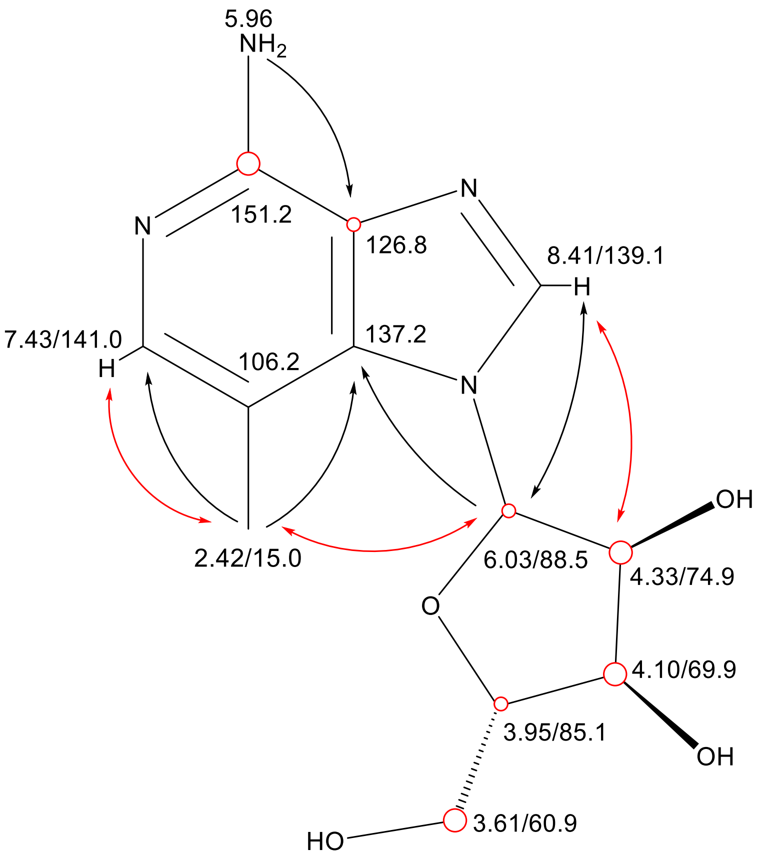

Figure S10. Scheme S2, #3 resonance assignments (^{1}H/^{13}C ppm) in DMSO-d_6 at 298K and pertinent NOE, HMBC, and isotope shift data. Larger (~10 Hz) isotope shifts are represented by larger circles.

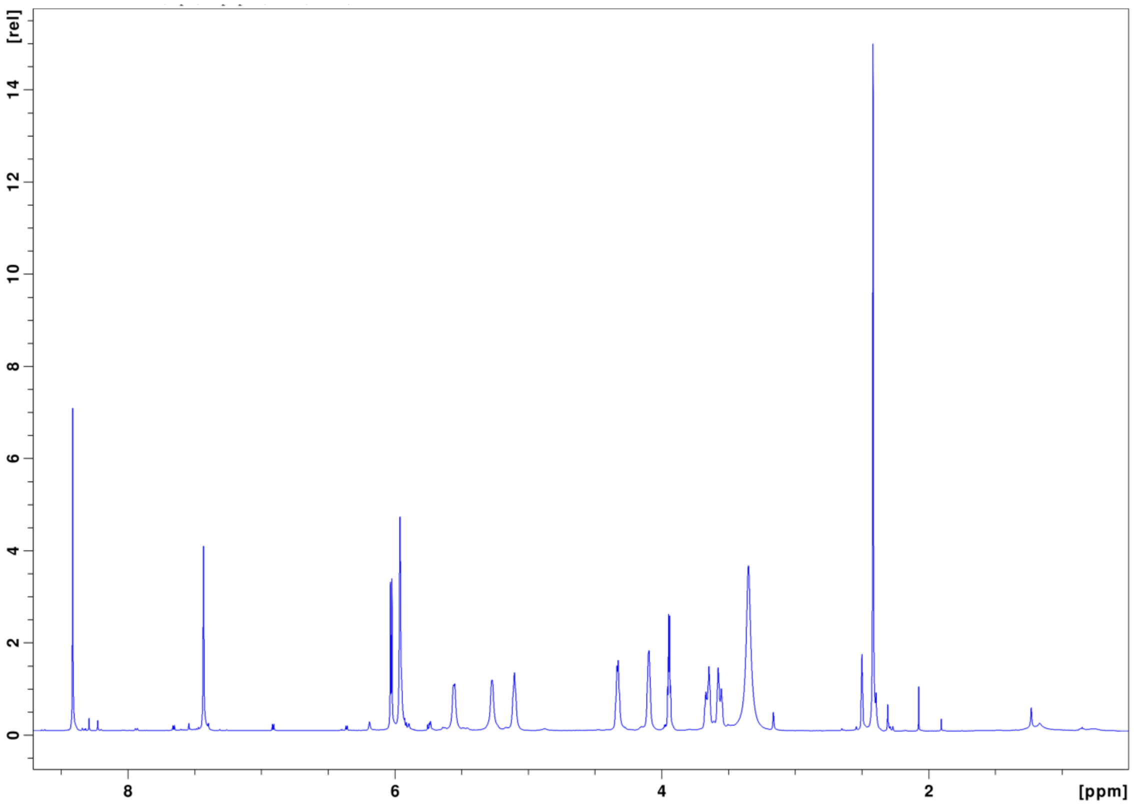

Figure S11. ^{1}H spectrum of Scheme S2, #3 in DMSO-d_6 at 298K. Assignments are in Figure S10.

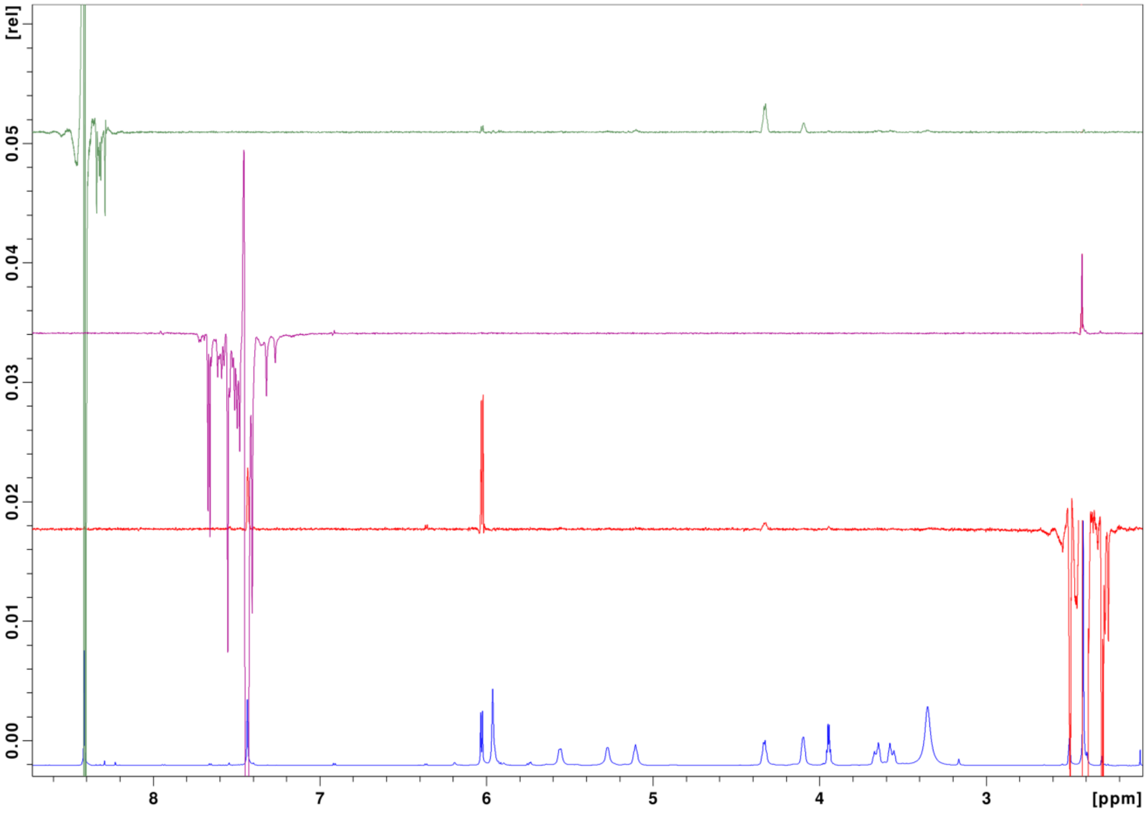

Figure S12. 1D-dpfgse spectra at 298K. The blue spectrum is for reference. The green dpfgse spectrum selectively inverts the 8.42 ppm ^{1}H and shows the ribose 4.33 ppm ^{1}H is nearest it. The purple spectrum selects the 7.44 ppm purine ^{1}H, showing only the methy near. The red dpfgse spectrum shows that the 6.0 ppm ribose ^{1}H is near the methyl.

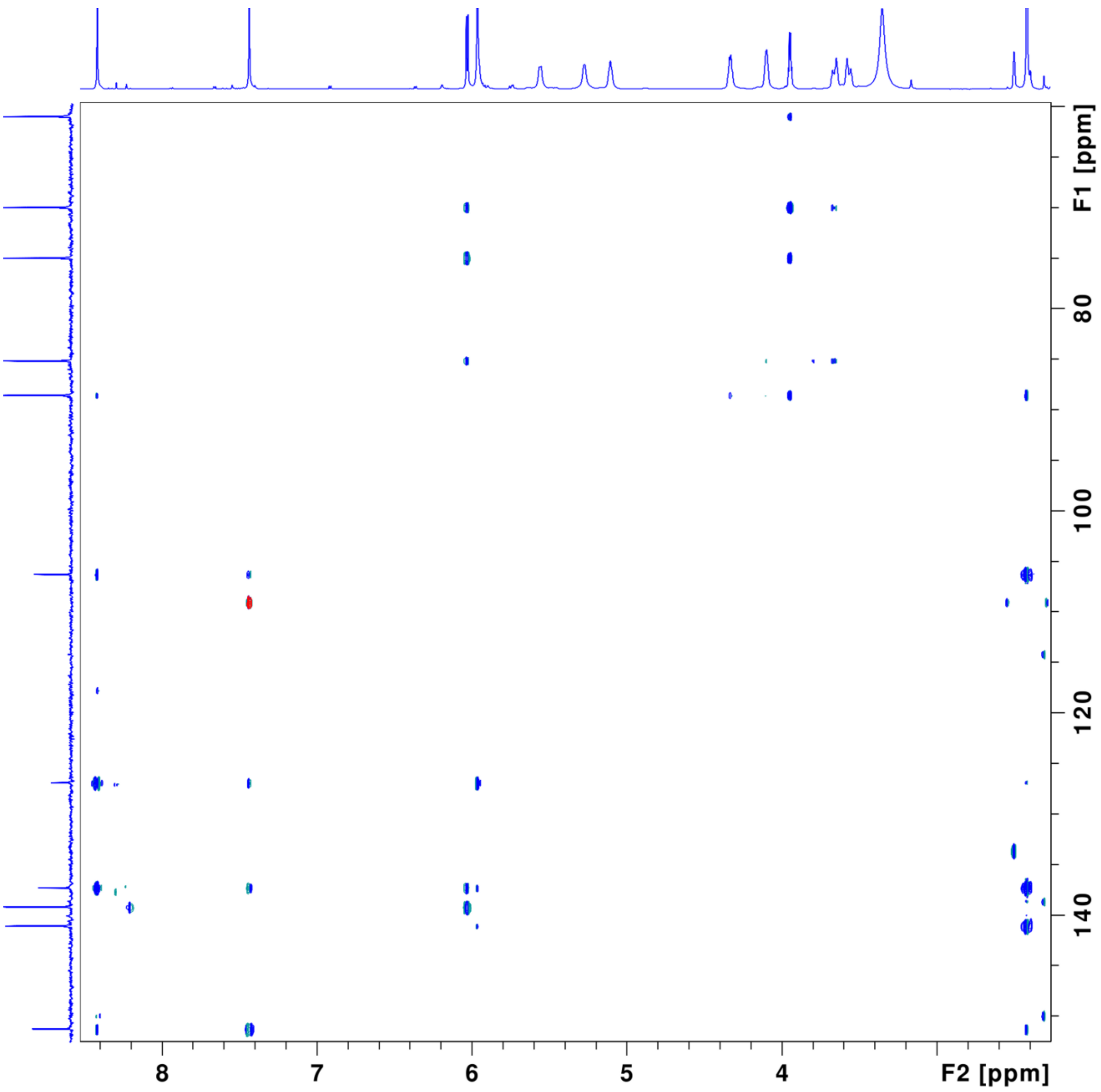

Figure S13. HMBC spectrum of Scheme S2, #3 at 298K optimized for a 4 Hz coupling. The red correlation is aliased from the methyl at 15 ppm.

Supplementary references

1 G. Lebon, T. Warne, P. C. Edwards, K. Bennett, C. J. Langmead, A. G. W. Leslie and C. G. Tate, *Nature*, 2011, **474**, 521–525.
2 B. B. Fredholm, E. Irenius, B. Kull and G. Schulte, *Biochem. Pharmacol.*, 2001, **61**, 443–448.
3 D. Rodríguez, S. Chakraborty, E. Warnick, S. Crane, Z. G. Gao, R. O'Connor, K. A. Jacobson and J. Carlsson, *ACS Chem. Biol.*, 2016, **11**, 2763–2772.
4 D. Rodríguez, X. Bello and H. Gutiérrez-De-Terán, *Mol. Inform.*, 2012, **31**, 334–341.
5 H. J. C. Berendsen, D. van der Spoel and R. van Drunen, *Comput. Phys. Commun.*, 1995, **91**, 43–56.
6 W. L. Jorgensen, D. S. Maxwell and J. Tirado-Rives, *J. Am. Chem. Soc.*, 1996, **118**, 11225–11236.
7 O. Berger, O. Edholm and F. Jähnig, *Biophys. J.*, 1997, **72**, 2002–2013.
8 W. L. Jorgensen, J. Chandrasekhar, J. D. Madura, R. W. Impey and M. L. Klein, *J. Chem. Phys.*, 1983, **79**, 926–935.
9 J. Marelius, K. Kolmodin, I. Feierberg and J. Åqvist, *J. Mol. Graph. Model.*, 1998, **16**, 213–225.
10 M. J. Robertson, J. Tirado-Rives and W. L. Jorgensen, *J. Chem. Theory Comput.*, 2015, **11**, 3499–3509.
11 L. S. Dodda, I. C. De Vaca, J. Tirado-Rives and W. L. Jorgensen, *Nucleic Acids Res.*, 2017, **45**, 331–336.
12 G. King and A. Warshel, *J. Chem. Phys.*, 1989, **91**, 3647–3661.
13 J. P. Ryckaert, G. Ciccotti and H. J. C. Berendsen, *J. Comput. Phys.*, 1977, **23**, 327–341.
14 F. S. Lee and A. Warshel, *J. Chem. Phys.*, 1992, **97**, 3100–3107.
15 T. C. Beutler, A. E. Mark, R. C. van Schaik, P. R. Gerber and W. F. van Gunsteren, *Chem. Phys. Lett.*, 1994, **222**, 529–539.
16 P. Matricon, A. Ranganathan, E. Warnick, Z. G. Gao, A. Rudling, C. Lambertucci, G. Marucci, A. Ezzati, M. Jaiteh, D. Dal Ben, K. A. Jacobson and J. Carlsson, *Sci. Rep.*, 2017, **7**, 6398.
17 K. Haider, A. Cruz, S. Ramsey, M. K. Gilson and T. Kurtzman, *J. Chem. Theory Comput.*, 2018, **14**, 418–425.
18 T. Young, R. Abel, B. Kim, B. J. Berne and R. A. Friesner, *Proc. Natl. Acad. Sci. U. S. A.*, 2007, **104**, 808–813.
19 R. Abel, T. Young, R. Farid, B. J. Berne and R. A. Friesner, *J. Am. Chem. Soc.*, 2008, **130**, 2817–2831.
20 O. Trott and A. J. Olson, *J. Comput. Chem.*, 2010, **31**, 455–461.
21 R. A. Friesner, J. L. Banks, R. B. Murphy, T. A. Halgren, J. J. Klicic, D. T. Mainz, M. P. Repasky, E. H. Knoll, M. Shelley, J. K. Perry, D. E. Shaw, P. Francis and P. S. Shenkin, *J. Med. Chem.*, 2004, **47**, 1739–1749.
22 S. Paoletta, D. K. Tosh, A. Finley, E. T. Gizewski, S. M. Moss, Z. G. Gao, J. A. Auchampach, D. Salvemini and K. A. Jacobson, *J. Med. Chem.*, 2013, **56**, 5949-5963.
23 C. Yung-Chi and W. H. Prusoff, *Biochem. Pharmacol.*, 1973, **22**, 3099–3108.
24 G. Framski, Z. Gdaniec, M. Gdaniec and J. Boryski, *Tetrahedron*, 2006, **62**, 10123–10129.
25 M. D. Erlacher, K. Lang, B. Wotzel, R. Rieder, R. Micura and N. Polacek, *J. Am. Chem. Soc.*, 2006, **128**, 4453–4459.
